# Supplementary material for: Disability and quality of life assessment using WHODAS-12 items 2.0 and EQ-5D-5L in a rural area endemic for loiasis in the Republic of Congo: A population-based cross-sectional study (the MorLo project)
Source: PLoS Negl Trop Dis. 2025 Sep 15;19(9):e0013491. doi: 10.1371/journal.pntd.0013491 (PMC12449028; doi:10.1371/journal.pntd.0013491)
Supplement: S1 Text — (DOCX) [file pntd.0013491.s001.docx]

**S1 Text.** WHODAS 2.0 questionnaire.

***Read:*** “This survey is about the difficulties people may face because of their health condition.”

***Hand over card 1 and read:*** “By health condition, I mean illness or discomfort, or any other health problem that may be short-term or chronic, injury, mental or emotional problems, and problems related to alcohol or drugs.”

“I ask you to keep all your health problems in mind when answering the questions. When I ask you to tell me about any difficulties you have performing an activity, please think about:” (***show card 1***)

“Increased effort, discomfort or pain, slowness, a change in the way you perform these activities.”

“When answering the questions, I'd like you to think about the last 30 days. I'd also like you to respond by estimating the difficulty you had, on average, over the last 30 days, comparing it to the difficulty you'd have if you were doing this activity as usual.”

***Show card 2 and say:*** “Use this rating scale when you tell me about your difficulties.”

***Read the scale aloud:*** “None, Mild, Moderate, Severe, Extreme or Can't do.”

***Make sure the respondent can always see the cards throughout the interview.***

| In the last 30 days, how much difficulty did you have in: | | None | Mild | Moderate | Severe | Extreme |
| --- | --- | --- | --- | --- | --- | --- |
| 1 | Standing for long periods such as 30 minutes? | 0 | 1 | 2 | 3 | 4 |
| 2 | Taking care or your household responsibilities? | 0 | 1 | 2 | 3 | 4 |
| 3 | Learning a new task, for example, learning how to get to a new place? | 0 | 1 | 2 | 3 | 4 |
| 4 | Joining in community activities (for example, festivities, religious or other activities) in the same way as anyone else can? | 0 | 1 | 2 | 3 | 4 |
| 5 | How much have you been emotionally affected by your health problems? | 0 | 1 | 2 | 3 | 4 |
| 6 | Concentrating on doing something for ten minutes? | 0 | 1 | 2 | 3 | 4 |
| 7 | Walking a long distance such as a kilometer? | 0 | 1 | 2 | 3 | 4 |
| 8 | Washing your whole body? | 0 | 1 | 2 | 3 | 4 |
| 9 | Getting dressed? | 0 | 1 | 2 | 3 | 4 |
| 10 | Dealing with people you do not know? | 0 | 1 | 2 | 3 | 4 |
| 11 | Maintaining a friendship? | 0 | 1 | 2 | 3 | 4 |
| 12 | Your day-to-day work? | 0 | 1 | 2 | 3 | 4 |
| H1 | In total, over the last 30 days, how many days did you experience these difficulties? | | | ***Number of days:*** | | |
| H2 | During the last 30 days, for how many days were you unable to carry out your usual activities or work due to your state of health? | | | ***Number of days:*** | | |
| H3 | During the last 30 days, excluding days when you were totally incapacitated, for how long did you reduce or curtail your usual activities or work because of your state of health? | | | ***Number of days:*** | | |
