# Supplementary material for: Disability and quality of life assessment using WHODAS-12 items 2.0 and EQ-5D-5L in a rural area endemic for loiasis in the Republic of Congo: A population-based cross-sectional study (the MorLo project)
Source: PLoS Negl Trop Dis. 2025 Sep 15;19(9):e0013491. doi: 10.1371/journal.pntd.0013491 (PMC12449028; doi:10.1371/journal.pntd.0013491)
Supplement: S2 Text — (DOCX) [file pntd.0013491.s002.docx]

**S2 Text.** EQ-5D-5L questionnaire.

| We're trying to gather your thoughts about your health. I'll be explaining things as I go along, but don't hesitate to interrupt me if you don't understand something or if things don't seem clear to you. There are no right or wrong answers. We're only interested in your personal opinion. |
| --- |
| First, I'm going to read you some questions. Each question offers a choice of five answers. Please tell me which answer best describes your health TODAY. |
| Please choose no more than one answer for each group of questions. |
| (Evaluator's note: First, read the five choices for each question. Then ask the interviewee which one applies to him/her. Repeat the question and propositions if necessary. Tick the appropriate box under each heading. You may need to remind the interviewee regularly that it's all about TODAY). |

| First of all, I'd like to ask you about MOBILITY. Would you say that: | |
| --- | --- |
| 1. you have no problems getting around on foot? | ❑ |
| 2. you have slight problems getting around on foot? | ❑ |
| 3. you have moderate problems getting around on foot? | ❑ |
| 4. you have severe problems getting around on foot? | ❑ |
| 5. you are unable to get around on foot? | ❑ |
| Next, I'd like to ask you about AUTONOMY. Would you say that: | |
| 1. you have no problems washing or dressing yourself? | ❑ |
| 2. you have slight problems washing or dressing yourself? | ❑ |
| 3. you have moderate problems washing or dressing yourself? | ❑ |
| 4. you have severe problems washing or dressing yourself? | ❑ |
| 5. you are unable to wash or dress yourself? | ❑ |
| Next, I'd like to ask you about CURRENT ACTIVITIES, for example, work, study, housework, family or leisure activities. Would you say that: | |
| 1. you have no problems in carrying out your daily activities? | ❑ |
| 2. you have slight problems performing your daily activities? | ❑ |
| 3. you have moderate problems in carrying out your day-to-day activities? | ❑ |
| 4. you have severe problems performing your daily activities? | ❑ |
| 5. you are unable to perform your daily activities? | ❑ |
| Next, I'd like to ask you about PAIN(S) or discomfort. | |
| Would you say that: | ❑ |
| 1. you have no pain or discomfort? | ❑ |
| 2. you have slight pain or discomfort? | ❑ |
| 3. you have moderate pain or discomfort? | ❑ |
| 4. you have severe pain or discomfort? | ❑ |
| Finally, I'd like to ask you about ANXIETY or DEPRESSION. | |
| Would you say that: | ❑ |
| 1. you are neither anxious nor depressed? | ❑ |
| 2. you are slightly anxious or depressed? | ❑ |
| 3. you are moderately anxious or depressed? | ❑ |
| 4. you are severely anxious or depressed? | ❑ |

| EQ-5D EVA |
| --- |
| - Now I'd like to ask you how good or bad your health is TODAY. |
| - I'd like you to imagine a vertical line graduated from 0 to 100. |
| (Note to evaluator: if the interview is face-to-face, please show the EVA line to the interviewee). |
| - 100 at the top of the line indicates the best health you can imagine. |
| - 0 at the bottom of the line indicates the worst health you can imagine. |
| - Now I'd like you to tell me where on this line you would place your health TODAY. |
| (Evaluator's note: place the point on the line indicating the respondent's health today. Now, please enter the number you indicated on the line in the box below). |
