# Supplementary material for: Disability and quality of life assessment using WHODAS-12 items 2.0 and EQ-5D-5L in a rural area endemic for loiasis in the Republic of Congo: A population-based cross-sectional study (the MorLo project)
Source: PLoS Negl Trop Dis. 2025 Sep 15;19(9):e0013491. doi: 10.1371/journal.pntd.0013491 (PMC12449028; doi:10.1371/journal.pntd.0013491)
Supplement: S1 Table — (DOCX) [file pntd.0013491.s003.docx]

**S1 Table.** Disabilities for the 6 domains from the WHODAS 2.0 questionnaire.

|  |  |  | Cognitive/100 | | Mobility/100 | | Selfcare/100 | | Social/100 | | Household/100 | | Participation/100 | |
| --- | --- | --- | --- | --- | --- | --- | --- | --- | --- | --- | --- | --- | --- | --- |
|  |  | N | Mean | SD | Mean | SD | Mean | SD | Mean | SD | Mean | SD | Mean | SD |
| Total |  | 987 | 31.9 | 30.1 | 37.7 | 30.3 | 5.6 | 14.2 | 6.0 | 11.7 | 40.5 | 27.4 | 30.3 | 27.3 |
| Sex | Female | 370 | 39.2 | 30.4 | 49.3 | 29.1 | 7.8 | 17.1 | 7.7 | 13.2 | 49.4 | 26.0 | 37.6 | 27.4 |
|  | Male | 617 | 27.5 | 29.0 | 30.8 | 28.9 | 4.2 | 11.9 | 4.9 | 10.7 | 35.2 | 26.9 | 25.8 | 26.3 |
| Age (y.o.) | 18-28 | 86 | 18.0 | 26.5 | 18.8 | 23.6 | 2.0 | 8.5 | 5.1 | 9.5 | 25.4 | 24.4 | 20.5 | 24.8 |
|  | 29-38 | 137 | 24.3 | 27.6 | 29.6 | 27.4 | 3.6 | 11.3 | 7.5 | 13.3 | 31.8 | 25.1 | 23.8 | 24.2 |
|  | 39-48 | 191 | 28.1 | 28.9 | 32.5 | 28.0 | 5.3 | 13.0 | 5.2 | 9.9 | 36.3 | 26.1 | 26.7 | 26.0 |
|  | 49-58 | 253 | 28.7 | 28.8 | 34.4 | 29.4 | 4.5 | 12.2 | 4.7 | 10.2 | 39.1 | 25.4 | 27.5 | 26.0 |
|  | 59-68 | 202 | 39.6 | 29.8 | 47.3 | 29.0 | 7.4 | 17.0 | 7.2 | 13.1 | 48.6 | 27.8 | 35.8 | 27.7 |
|  | >68 | 118 | 50.6 | 29.0 | 60.2 | 28.5 | 10.0 | 18.9 | 7.0 | 14.3 | 57.6 | 25.5 | 47.2 | 27.9 |
| Eye worm episodes | 0 | 423 | 29.4 | 30.0 | 34.5 | 30.6 | 5.2 | 14.2 | 6.0 | 12.2 | 39.1 | 28.1 | 28.5 | 27.7 |
|  | 1-5 | 172 | 31.6 | 30.3 | 36.8 | 29.4 | 5.9 | 14.9 | 4.8 | 10.0 | 38.9 | 26.2 | 29.3 | 27.1 |
|  | 6-10 | 140 | 34.5 | 28.4 | 43.1 | 28.8 | 4.2 | 10.9 | 4.8 | 9.2 | 42.7 | 26.0 | 33.6 | 25.7 |
|  | >10 | 79 | 36.1 | 28.4 | 44.8 | 29.8 | 5.5 | 13.7 | 9.3 | 12.7 | 44.6 | 26.1 | 34.2 | 24.8 |
|  | AMD* | 173 | 34.3 | 31.8 | 38.9 | 31.1 | 7.2 | 16.0 | 6.6 | 13.4 | 41.8 | 28.5 | 31.1 | 28.5 |
| Calabar episodes | 0 | 568 | 31.2 | 30.0 | 36.3 | 30.7 | 5.5 | 14.7 | 5.7 | 11.6 | 39.8 | 27.7 | 29.3 | 27.6 |
|  | 1-5 | 112 | 32.4 | 30.0 | 39.8 | 28.5 | 5.0 | 12.0 | 5.8 | 10.3 | 43.0 | 25.0 | 32.4 | 25.5 |
|  | 6-10 | 88 | 32.2 | 29.4 | 39.7 | 30.0 | 4.4 | 11.6 | 6.7 | 11.5 | 39.9 | 28.4 | 33.0 | 25.9 |
|  | >10 | 47 | 31.6 | 26.7 | 40.4 | 26.6 | 3.2 | 8.0 | 6.6 | 10.7 | 40.2 | 24.2 | 29.8 | 26.0 |
|  | AMD | 172 | 34.0 | 31.7 | 39.3 | 31.3 | 7.4 | 16.1 | 6.5 | 13.3 | 41.7 | 28.7 | 31.0 | 28.5 |
| *Loa* MFD (mf/mL) | 0 | 638 | 32.2 | 30.2 | 37.6 | 30.5 | 5.8 | 14.6 | 5.8 | 11.7 | 40.9 | 27.5 | 30.7 | 27.5 |
|  | 1-7,999 | 252 | 32.4 | 30.5 | 38.5 | 30.3 | 5.2 | 14.4 | 6.0 | 12.0 | 40.5 | 27.5 | 30.5 | 27.2 |
|  | -19,999 | 65 | 28.7 | 29.5 | 34.2 | 30.7 | 5.6 | 10.1 | 6.0 | 11.1 | 35.4 | 25.9 | 28.8 | 27.8 |
|  | >19,999 | 32 | 28.1 | 26.4 | 40.2 | 26.3 | 4.3 | 11.3 | 9.0 | 11.6 | 43.0 | 28.2 | 22.7 | 22.3 |
| *Loa* RDT (Intensity)* | 0 | 59 | 30.1 | 27.7 | 40.5 | 27.3 | 4.4 | 12.9 | 5.9 | 11.2 | 39.2 | 27.9 | 27.8 | 25.8 |
|  | 1-2 | 102 | 30.9 | 31.0 | 36.4 | 31.7 | 5.3 | 12.0 | 6.0 | 13.0 | 40.4 | 28.4 | 28.9 | 28.6 |
|  | 3-4 | 410 | 34.0 | 30.1 | 40.4 | 30.7 | 6.6 | 15.4 | 6.9 | 12.7 | 42.5 | 27.7 | 31.1 | 26.8 |
|  | 5-6 | 354 | 29.4 | 29.5 | 34.5 | 29.6 | 4.3 | 12.6 | 5.2 | 10.5 | 39.1 | 26.8 | 29.3 | 27.0 |
|  | >6 | 44 | 34.1 | 33.5 | 38.1 | 31.4 | 9.7 | 18.8 | 4.8 | 8.6 | 39.5 | 27.5 | 33.8 | 30.0 |
| Eosinophilia (× 10^9^ cells/L) | ≤2 | 766 | 31.0 | 29.6 | 36.7 | 30.2 | 5.3 | 13.9 | 5.9 | 11.8 | 39.9 | 27.3 | 29.4 | 26.8 |
|  | >2 | 148 | 30.1 | 31.0 | 39.1 | 30.5 | 6.9 | 15.6 | 6.4 | 10.6 | 40.9 | 27.5 | 28.7 | 26.8 |
|  | AMD | 73 | 44.9 | 30.6 | 45.5 | 30.2 | 5.1 | 13.9 | 5.5 | 13.5 | 46.1 | 28.3 | 43.2 | 30.0 |
| Main occupation | Other | 211 | 31.5 | 31.8 | 36.4 | 32.8 | 6.4 | 16.4 | 7.5 | 15.1 | 37.6 | 30.8 | 30.4 | 29.3 |
|  | Farmer | 776 | 32.0 | 29.6 | 38.1 | 29.7 | 5.3 | 13.5 | 5.6 | 10.6 | 41.3 | 26.4 | 30.2 | 26.7 |
| Marital status | Couple | 627 | 28.6 | 29.1 | 34.8 | 29.4 | 4.8 | 13.2 | 5.4 | 10.8 | 37.8 | 26.8 | 27.5 | 26.1 |
|  | Single | 360 | 37.7 | 30.9 | 42.7 | 31.4 | 6.8 | 15.8 | 7.0 | 13.2 | 45.2 | 27.8 | 35.2 | 28.6 |
| SCD status* | HbAA | 757 | 32.4 | 30.2 | 38.2 | 30.3 | 5.5 | 13.9 | 5.8 | 11.5 | 41.1 | 27.7 | 30.9 | 27.4 |
|  | HbAS | 219 | 30.3 | 29.6 | 36.0 | 30.2 | 5.4 | 14.7 | 6.9 | 12.8 | 38.6 | 26.2 | 27.7 | 26.5 |
| Tobacco use* | No | 797 | 32.7 | 30.3 | 39.0 | 30.5 | 5.8 | 14.5 | 6.2 | 12.0 | 41.4 | 27.6 | 30.9 | 27.6 |
|  | Yes | 182 | 29.3 | 29.1 | 31.8 | 28.7 | 4.8 | 12.8 | 5.2 | 10.6 | 37.2 | 26.5 | 28.3 | 26.2 |
| *Ascaris lumbricoides* (epg) | 0 | 437 | 29.6 | 29.4 | 36.1 | 29.6 | 5.1 | 14.2 | 5.6 | 10.3 | 39.0 | 26.2 | 28.8 | 26.0 |
|  | 1-1,000 | 210 | 31.1 | 29.9 | 35.9 | 29.9 | 4.6 | 13.4 | 6.1 | 12.8 | 40.1 | 27.4 | 29.4 | 28.0 |
|  | >1,000 | 121 | 36.6 | 29.6 | 42.8 | 31.3 | 7.1 | 14.2 | 6.6 | 12.9 | 43.8 | 28.9 | 33.3 | 28.0 |
|  | AMD | 219 | 34.7 | 31.5 | 39.9 | 31.4 | 6.5 | 14.7 | 6.3 | 12.8 | 42.1 | 28.8 | 32.3 | 28.6 |
| *Trichuris trichiura* infection | No | 564 | 30.1 | 29.6 | 37.1 | 30.2 | 5.4 | 14.7 | 5.7 | 10.9 | 39.4 | 27.0 | 29.0 | 26.3 |
|  | Yes | 206 | 33.9 | 29.8 | 37.0 | 29.6 | 5.1 | 12.1 | 6.6 | 13.0 | 41.6 | 27.1 | 31.8 | 28.3 |
|  | AMD | 217 | 34.7 | 31.5 | 40.1 | 31.4 | 6.5 | 14.8 | 6.1 | 12.7 | 42.2 | 28.9 | 32.3 | 28.7 |

* Other variables: total (4 missing data), RDT *Loa* (18), Tobacco (8), SCD (7 invalid results and 4 absent/missing data - AMD).

SD: Standard deviation. MD: Missing data. MFD: Microfilarial density. mf/mL: microfilariae per milliliter of blood. RDT: Rapid diagnostic test. SCD: Sickle cell disease. epg: eggs per gram of stool
