# Supplementary material for: Disability and quality of life assessment using WHODAS-12 items 2.0 and EQ-5D-5L in a rural area endemic for loiasis in the Republic of Congo: A population-based cross-sectional study (the MorLo project)
Source: PLoS Negl Trop Dis. 2025 Sep 15;19(9):e0013491. doi: 10.1371/journal.pntd.0013491 (PMC12449028; doi:10.1371/journal.pntd.0013491)
Supplement: S2 Table — (DOCX) [file pntd.0013491.s004.docx]

**S2 Table.** Descriptive results for WHODAS 2.0 questionnaire for the number of days impaired during the last month.

|  |  | Mild disability | | Full disability | | Partial disability | |
| --- | --- | --- | --- | --- | --- | --- | --- |
|  |  | Mean | SD | Mean | SD | Mean | SD |
| Total |  | 17.6 | 12.3 | 10.8 | 12.6 | 5.2 | 10.0 |
| Sex | Female | 21.2 | 11.3 | 13.9 | 12.8 | 6.5 | 11.0 |
|  | Male | 15.4 | 12.4 | 8.9 | 12.1 | 4.4 | 9.3 |
| Age (y.o.) | 18-28 | 10.2 | 11.3 | 4.3 | 8.4 | 3.2 | 8.1 |
|  | 29-38 | 13.1 | 11.9 | 7.2 | 10.8 | 5.0 | 9.7 |
|  | 39-48 | 15.1 | 12.5 | 8.8 | 12.0 | 4.6 | 9.6 |
|  | 49-58 | 16.8 | 11.9 | 10.2 | 12.3 | 5.6 | 10.0 |
|  | 59-68 | 22.3 | 10.9 | 13.4 | 13.1 | 5.4 | 10.2 |
|  | >68 | 25.8 | 8.8 | 19.5 | 12.3 | 6.9 | 11.6 |
| Eye worm episodes | 0 | 16.6 | 12.6 | 9.9 | 12.5 | 4.9 | 9.7 |
|  | 1-5 | 17.6 | 12.4 | 10.1 | 12.2 | 4.8 | 9.5 |
|  | 6-10 | 19.3 | 11.9 | 12.7 | 12.9 | 6.6 | 11.4 |
|  | >10 | 20.3 | 10.6 | 12.6 | 13.0 | 6.3 | 11.1 |
|  | AMD* | 17.4 | 12.3 | 11.3 | 12.7 | 4.7 | 9.5 |
| Calabar episodes | 0 | 17.7 | 12.6 | 10.5 | 12.7 | 5.3 | 10.1 |
|  | 1-5 | 18.6 | 11.7 | 12.6 | 12.7 | 4.3 | 9.0 |
|  | 6-10 | 16.0 | 12.0 | 10.1 | 12.1 | 5.9 | 10.7 |
|  | >10 | 17.8 | 11.4 | 9.6 | 12.5 | 7.0 | 11.7 |
|  | AMD | 17.3 | 12.4 | 11.2 | 12.6 | 4.7 | 9.5 |
| *Loa* MFD (mf/mL) | 0 | 17.7 | 12.4 | 10.9 | 12.8 | 5.3 | 10.0 |
|  | 1-7,999 | 18.1 | 12.3 | 11.1 | 12.4 | 5.2 | 10.1 |
|  | 8,000-19,999 | 15.0 | 12.3 | 9.0 | 12.2 | 4.6 | 9.8 |
|  | >19,999 | 17.2 | 11.0 | 9.8 | 11.5 | 6.0 | 9.8 |
| *Loa* RDT (Intensity)* | 0 | 19.3 | 11.8 | 11.5 | 12.8 | 4.6 | 9.2 |
|  | 1-2 | 18.6 | 12.1 | 11.0 | 12.9 | 6.0 | 11.1 |
|  | 3-4 | 17.9 | 12.3 | 11.5 | 12.8 | 6.0 | 10.7 |
|  | 5-6 | 16.9 | 12.3 | 10.1 | 12.2 | 4.4 | 9.0 |
|  | >6 | 15.6 | 12.8 | 10.6 | 12.7 | 4.6 | 9.7 |
| Eosinophilia (× 10^9^ cells/L) | ≤2 | 17.6 | 12.2 | 10.9 | 12.6 | 5.3 | 10.1 |
|  | >2 | 17.5 | 12.5 | 11.3 | 12.5 | 4.9 | 9.8 |
|  | AMD | 18.0 | 12.9 | 8.7 | 12.4 | 4.8 | 10.0 |
| Main occupation | Other | 16.5 | 12.8 | 11.6 | 13.5 | 4.8 | 10.0 |
|  | Farmer | 17.9 | 12.2 | 10.6 | 12.3 | 5.3 | 10.0 |
| Marital status | As a couple | 16.6 | 12.2 | 9.0 | 11.8 | 4.3 | 8.9 |
|  | Single | 19.4 | 12.4 | 13.9 | 13.3 | 6.9 | 11.5 |
| Sickle celle disease status* | HbAA | 17.8 | 12.4 | 10.9 | 12.7 | 5.1 | 10.0 |
|  | HbAS | 17.1 | 12.2 | 10.4 | 12.1 | 5.5 | 10.0 |
| Tobacco use* | No | 17.9 | 12.4 | 10.8 | 12.6 | 5.2 | 10.1 |
|  | Yes | 16.2 | 12.2 | 10.9 | 12.5 | 5.2 | 9.8 |
| *Ascaris lumbricoides* (epg) | 0 | 17.1 | 12.1 | 10.0 | 12.1 | 5.3 | 10.1 |
|  | 1-1,000 | 17.4 | 12.5 | 10.0 | 12.4 | 4.6 | 9.4 |
|  | >1,000 | 19.7 | 12.7 | 13.6 | 14.0 | 7.3 | 11.8 |
|  | AMD | 17.7 | 12.3 | 11.6 | 12.8 | 4.5 | 9.3 |
| *Trichuris trichiura* infection | No | 17.3 | 12.3 | 10.1 | 12.3 | 5.3 | 10.1 |
|  | Yes | 18.4 | 12.4 | 11.9 | 13.0 | 5.9 | 10.7 |
|  | AMD | 17.6 | 12.3 | 11.6 | 12.7 | 4.5 | 9.1 |
|  | | | | | | | |

* missing data. Other variables: total (4 absent/missing data - AMD), RDT *Loa* (18), Tobacco (8), Sickle cell disease (7 invalid results and 4 missing data).

Mild disability (H1 question); Full disability (H2 question); Partial disability (H3 question).

SD: standard deviation. MFD: microfilarial density. mf/mL: microfilariae per milliliter of blood. RDT: Rapid diagnostic test. SCD: sickle cell disease. epg: eggs per gram of stool
