# Supplementary material for: Disability and quality of life assessment using WHODAS-12 items 2.0 and EQ-5D-5L in a rural area endemic for loiasis in the Republic of Congo: A population-based cross-sectional study (the MorLo project)
Source: PLoS Negl Trop Dis. 2025 Sep 15;19(9):e0013491. doi: 10.1371/journal.pntd.0013491 (PMC12449028; doi:10.1371/journal.pntd.0013491)
Supplement: S3 Table — (DOCX) [file pntd.0013491.s005.docx]

**S3 Table.** Saturated multivariable analyses on global WHODAS 2.0 score and the related six domains.

|  |  | Global WHODAS score | | Mobility | | Selfcare | | Household | |
| --- | --- | --- | --- | --- | --- | --- | --- | --- | --- |
|  |  | aIRR [95% CI] | p | aIRR [95% CI] | p | aIRR [95% CI] | p | aIRR [95% CI] | p |
| Sex (Ref: female) | Male | 0.81 [0.71, 0.93] | 0.003 | 0.74 [0.65, 0.84] | <0.001 | 0.72 [0.45, 1.16] | 0.180 | 0.86 [0.78, 0.94] | 0.002 |
| Age (Ref: 18-28 y.o.) | 29-38 | 1.39 [1.08, 1.79] | 0.011 | 1.70 [1.30, 2.24] | <0.001 | 1.59 [0.58, 4.39] | 0.371 | 1.28 [1.04, 1.58] | 0.021 |
|  | 39-48 | 1.46 [1.14, 1.86] | 0.002 | 1.73 [1.33, 2.25] | <0.001 | 2.17 [0.81, 5.79] | 0.123 | 1.42 [1.16, 1.74] | 0.001 |
|  | 49-58 | 1.57 [1.23, 2.00] | <0.001 | 1.83 [1.41, 2.37] | <0.001 | 2.30 [0.90, 5.90] | 0.083 | 1.57 [1.28, 1.91] | <0.001 |
|  | 59-68 | 1.79 [1.39, 2.31] | <0.001 | 2.19 [1.67, 2.86] | <0.001 | 3.00 [1.09, 8.22] | 0.033 | 1.68 [1.36, 2.06] | <0.001 |
|  | >68 | 2.05 [1.55, 2.70] | <0.001 | 2.60 [1.97, 3.44] | <0.001 | 3.88 [1.33, 11.26] | 0.013 | 1.85 [1.49, 2.29] | <0.001 |
| Eye worm episodes (Ref: 0) | 1-5 | 1.00 [0.85, 1.18] | 0.967 | 1.02 [0.87, 1.19] | 0.846 | 1.18 [0.65, 2.12] | 0.588 | 0.95 [0.84, 1.07] | 0.406 |
|  | 6-10 | 1.11 [0.93, 1.33] | 0.260 | 1.18 [1.00, 1.39] | 0.050 | 0.79 [0.40, 1.56] | 0.501 | 1.03 [0.91, 1.17] | 0.648 |
|  | >10 | 1.32 [1.05, 1.67] | 0.019 | 1.27 [1.02, 1.57] | 0.029 | 1.62 [0.72, 3.68] | 0.246 | 1.16 [0.98, 1.38] | 0.079 |
|  | AMD* | 1.03 [0.53, 2.01] | 0.922 | 0.78 [0.41, 1.48] | 0.440 | 0.58 [0.02, 21.35] | 0.764 | 1.01 [0.63, 1.63] | 0.965 |
| Calabar swelling episodes (Ref: 0) | 1-5 | 1.06 [0.88, 1.27] | 0.552 | 1.10 [0.92, 1.30] | 0.286 | 0.80 [0.40, 1.60] | 0.522 | 1.08 [0.95, 1.24] | 0.234 |
|  | 6-10 | 1.03 [0.83, 1.27] | 0.795 | 1.03 [0.84, 1.25] | 0.796 | 0.98 [0.44, 2.16] | 0.960 | 0.97 [0.83, 1.13] | 0.674 |
|  | >10 | 0.97 [0.73, 1.30] | 0.843 | 1.09 [0.83, 1.43] | 0.519 | 0.43 [0.14, 1.35] | 0.149 | 0.98 [0.79, 1.21] | 0.839 |
|  | AMD | 1.26 [0.67, 2.38] | 0.471 | 1.61 [0.88, 2.95] | 0.123 | 5.53 [0.16, 187.90] | 0.342 | 1.14 [0.72, 1.81] | 0.565 |
| *Loa* RDT (Intensity) (Ref: 0) | 1-2 | 1.06 [0.80, 1.41] | 0.673 | 0.97 [0.75, 1.26] | 0.821 | 1.46 [0.52, 4.13] | 0.472 | 1.10 [0.89, 1.36] | 0.363 |
|  | 3-4 | 1.14 [0.90, 1.46] | 0.280 | 1.06 [0.85, 1.33] | 0.583 | 1.64 [0.67, 4.01] | 0.278 | 1.13 [0.95, 1.35] | 0.170 |
|  | 5-6 | 1.06 [0.83, 1.35] | 0.659 | 0.98 [0.78, 1.23] | 0.879 | 1.06 [0.43, 2.62] | 0.902 | 1.10 [0.91, 1.31] | 0.329 |
|  | >6 | 1.21 [0.86, 1.72] | 0.278 | 1.13 [0.82, 1.56] | 0.458 | 2.54 [0.74, 8.65] | 0.136 | 1.15 [0.89, 1.48] | 0.299 |
| *Loa* MFD (mf/mL) (Ref: 0) | 1-7,999 | 1.02 [0.89, 1.16] | 0.775 | 1.06 [0.94, 1.20] | 0.355 | 0.87 [0.55, 1.39] | 0.571 | 1.00 [0.90, 1.10] | 0.936 |
|  | 8,000-19,999 | 0.84 [0.66, 1.06] | 0.139 | 0.85 [0.68, 1.07] | 0.169 | 0.97 [0.43, 2.21] | 0.944 | 0.84 [0.70, 1.00] | 0.052 |
|  | >19.999 | 1.06 [0.77, 1.45] | 0.722 | 1.20 [0.90, 1.60] | 0.205 | 0.79 [0.25, 2.43] | 0.677 | 1.11 [0.89, 1.39] | 0.354 |
| *Trichuris trichiura* infection (Ref: no) | Yes | 1.06 [0.91, 1.25] | 0.435 | 0.96 [0.83, 1.11] | 0.552 | 0.84 [0.47, 1.50] | 0.556 | 1.03 [0.91, 1.15] | 0.670 |
|  | AMD | 0.86 [0.25, 3.01] | 0.817 | 1.30 [0.36, 4.70] | 0.688 | 0.14 [0.00, 7.17] | 0.323 | 1.14 [0.42, 3.08] | 0.803 |
| *Ascaris lumbricoides* (epg) (Ref: 0) | 1-1,000 | 0.98 [0.84, 1.14] | 0.755 | 1.01 [0.88, 1.17] | 0.855 | 1.10 [0.63, 1.94] | 0.729 | 1.00 [0.90, 1.12] | 0.955 |
|  | >1,000 | 1.07 [0.88, 1.30] | 0.529 | 1.16 [0.97, 1.38] | 0.108 | 1.51 [0.75, 3.07] | 0.249 | 1.05 [0.91, 1.20] | 0.537 |
|  | AD | 1.07 [0.31, 3.63] | 0.917 | 0.76 [0.21, 2.68] | 0.668 | 4.05 [0.09, 192.67] | 0.478 | 0.85 [0.32, 2.28] | 0.752 |
| Eosinophilia (× 10^9^ cells/L) (Ref. ≤2) | >2 | 1.05 [0.90, 1.23] | 0.540 | 1.09 [0.94, 1.26] | 0.248 | 1.56 [0.87, 2.80] | 0.139 | 1.04 [0.93, 1.17] | 0.488 |
|  | AMD | 1.32 [1.05, 1.65] | 0.017 | 1.26 [1.02, 1.54] | 0.029 | 0.84 [0.37, 1.90] | 0.668 | 1.18 [1.01, 1.38] | 0.042 |
| Sickle cell status (Ref: HbAA) | HbAS | 0.94 [0.82, 1.07] | 0.355 | 0.93 [0.82, 1.05] | 0.253 | 0.94 [0.58, 1.53] | 0.797 | 0.93 [0.84, 1.03] | 0.144 |
| Body mass index (continuous) |  | 1.00 [0.98, 1.02] | 0.874 | 1.00 [0.99, 1.02] | 0.763 | 1.01 [0.95, 1.08] | 0.751 | 1.00 [0.98, 1.01] | 0.639 |
| Main occupation (Ref: other) | Farmer | 0.93 [0.80, 1.08] | 0.351 | 0.95 [0.82, 1.09] | 0.429 | 0.99 [0.58, 1.67] | 0.958 | 0.98 [0.88, 1.10] | 0.772 |
| Marital status (Ref: as a couple) | Alone | 1.14 [1.01, 1.29] | 0.040 | 1.04 [0.93, 1.17] | 0.472 | 1.30 [0.84, 2.01] | 0.242 | 1.10 [1.01, 1.21] | 0.031 |
| Years of schooling (continuous) |  | 0.96 [0.94, 0.98] | <0.001 | 0.96 [0.95, 0.98] | <0.001 | 0.96 [0.91, 1.02] | 0.233 | 0.96 [0.95, 0.98] | <0.001 |
| Tobacco use (Ref: no) | Yes | 1.01 [0.86, 1.18] | 0.908 | 1.01 [0.87, 1.18] | 0.872 | 1.00 [0.57, 1.76] | 0.999 | 1.02 [0.91, 1.15] | 0.704 |
| Mean blood pressure (continuous) |  | 1.00 [1.00, 1.00] | 0.836 | 1.00 [1.00, 1.00] | 0.471 | 1.00 [0.99, 1.01] | 0.898 | 1.00 [1.00, 1.00] | 0.437 |
|  |  | Cognitive |  | Social |  | Participation |  |  |  |
|  |  | aIRR [95% CI] | p | aIRR [95% CI] | p | aIRR [95% CI] | p |  |  |
| Sex (Ref: female) | Male | 0.93 [0.79, 1.10] | 0.426 | 0.57 [0.42, 0.78] | <0.001 | 0.84 [0.73, 0.96] | 0.014 |  |  |
| Age (Ref: 18-28 y.o.) | 29-38 | 1.48 [1.05, 2.08] | 0.024 | 1.47 [0.84, 2.57] | 0.177 | 1.23 [0.93, 1.64] | 0.147 |  |  |
|  | 39-48 | 1.67 [1.20, 2.31] | 0.002 | 0.96 [0.55, 1.68] | 0.889 | 1.32 [1.01, 1.74] | 0.044 |  |  |
|  | 49-58 | 1.78 [1.29, 2.46] | <0.001 | 0.82 [0.47, 1.42] | 0.473 | 1.38 [1.05, 1.81] | 0.020 |  |  |
|  | 59-68 | 2.09 [1.50, 2.93] | <0.001 | 1.18 [0.67, 2.09] | 0.572 | 1.57 [1.19, 2.08] | 0.002 |  |  |
|  | >68 | 2.32 [1.62, 3.31] | <0.001 | 0.98 [0.53, 1.82] | 0.948 | 1.86 [1.38, 2.50] | <0.001 |  |  |
| Eye worm episodes (Ref: 0) | 1-5 | 1.05 [0.86, 1.29] | 0.641 | 0.80 [0.55, 1.17] | 0.255 | 0.98 [0.82, 1.17] | 0.829 |  |  |
|  | 6-10 | 1.13 [0.91, 1.41] | 0.263 | 0.88 [0.58, 1.34] | 0.564 | 1.09 [0.90, 1.31] | 0.369 |  |  |
|  | >10 | 1.37 [1.02, 1.83] | 0.035 | 1.87 [1.14, 3.07] | 0.013 | 1.29 [1.01, 1.65] | 0.041 |  |  |
|  | AMD* | 1.22 [0.56, 2.66] | 0.612 | 2.14 [0.50, 9.18] | 0.307 | 0.89 [0.44, 1.80] | 0.749 |  |  |
| Calabar swelling episodes (Ref: 0) | 1-5 | 1.01 [0.80, 1.27] | 0.950 | 0.95 [0.62, 1.45] | 0.798 | 1.10 [0.91, 1.33] | 0.341 |  |  |
|  | 6-10 | 1.00 [0.77, 1.31] | 0.974 | 1.11 [0.70, 1.77] | 0.652 | 1.12 [0.91, 1.40] | 0.289 |  |  |
|  | >10 | 0.90 [0.63, 1.30] | 0.588 | 0.90 [0.47, 1.71] | 0.747 | 0.92 [0.67, 1.26] | 0.598 |  |  |
|  | AMD | 1.09 [0.52, 2.27] | 0.821 | 0.82 [0.19, 3.58] | 0.794 | 1.24 [0.64, 2.42] | 0.524 |  |  |
| *Loa* RDT (Intensity) (Ref: 0) | 1-2 | 1.18 [0.83, 1.69] | 0.361 | 0.99 [0.53, 1.87] | 0.977 | 1.08 [0.80, 1.47] | 0.600 |  |  |
|  | 3-4 | 1.22 [0.90, 1.65] | 0.203 | 1.28 [0.75, 2.19] | 0.368 | 1.14 [0.88, 1.47] | 0.324 |  |  |
|  | 5-6 | 1.12 [0.82, 1.52] | 0.475 | 0.95 [0.55, 1.65] | 0.849 | 1.15 [0.89, 1.49] | 0.296 |  |  |
|  | >6 | 1.32 [0.86, 2.03] | 0.204 | 0.95 [0.42, 2.12] | 0.899 | 1.37 [0.95, 1.97] | 0.088 |  |  |
| *Loa* MFD (mf/mL) (Ref: 0) | 1-7,999 | 1.03 [0.88, 1.21] | 0.719 | 0.98 [0.73, 1.31] | 0.872 | 1.02 [0.89, 1.18] | 0.732 |  |  |
|  | 8,000-19,999 | 0.81 [0.60, 1.09] | 0.159 | 1.00 [0.60, 1.69] | 0.988 | 0.90 [0.70, 1.16] | 0.413 |  |  |
|  | >19.999 | 0.98 [0.66, 1.45] | 0.918 | 1.80 [0.96, 3.40] | 0.069 | 0.81 [0.57, 1.15] | 0.247 |  |  |
| *Trichuris trichiura* infection (Ref: no) | Yes | 1.11 [0.92, 1.35] | 0.282 | 1.26 [0.89, 1.78] | 0.196 | 1.10 [0.93, 1.29] | 0.263 |  |  |
|  | AMD | 0.84 [0.18, 3.90] | 0.826 | 0.28 [0.03, 2.25] | 0.229 | 0.93 [0.26, 3.34] | 0.909 |  |  |
| *Ascaris lumbricoides* (epg) (Ref: 0) | 1-1,000 | 0.97 [0.80, 1.17] | 0.740 | 1.12 [0.80, 1.57] | 0.510 | 0.99 [0.84, 1.16] | 0.871 |  |  |
|  | >1,000 | 1.08 [0.85, 1.38] | 0.507 | 1.01 [0.65, 1.56] | 0.966 | 1.03 [0.84, 1.26] | 0.797 |  |  |
|  | AD | 1.12 [0.25, 5.03] | 0.882 | 2.39 [0.32, 18.02] | 0.398 | 1.10 [0.31, 3.87] | 0.881 |  |  |
| Eosinophilia (× 10^9^ cells/L) (Ref. ≤2) | >2 | 0.94 [0.77, 1.15] | 0.538 | 1.05 [0.74, 1.50] | 0.768 | 0.99 [0.83, 1.17] | 0.884 |  |  |
|  | AMD | 1.54 [1.17, 2.01] | 0.002 | 1.07 [0.65, 1.77] | 0.788 | 1.50 [1.20, 1.87] | <0.001 |  |  |
| Sickle cell status (Ref: HbAA) | HbAS | 0.94 [0.79, 1.11] | 0.451 | 1.17 [0.87, 1.56] | 0.303 | 0.89 [0.77, 1.03] | 0.116 |  |  |
| Body mass index (continuous) |  | 1.01 [0.98, 1.03] | 0.587 | 0.99 [0.95, 1.03] | 0.611 | 1.00 [0.98, 1.02] | 0.810 |  |  |
| Main occupation (Ref: other) | Farmer | 0.90 [0.75, 1.08] | 0.237 | 0.67 [0.49, 0.92] | 0.012 | 0.88 [0.75, 1.03] | 0.107 |  |  |
| Marital status (Ref: as a couple) | Alone | 1.24 [1.07, 1.44] | 0.005 | 1.09 [0.83, 1.43] | 0.524 | 1.17 [1.03, 1.33] | 0.017 |  |  |
| Years of schooling (continuous) |  | 0.95 [0.93, 0.96] | <0.001 | 1.00 [0.96, 1.04] | 0.999 | 0.96 [0.94, 0.98] | <0.001 |  |  |
| Tobacco use (Ref: no) | Yes | 0.99 [0.81, 1.20] | 0.902 | 1.05 [0.73, 1.50] | 0.805 | 1.04 [0.88, 1.22] | 0.668 |  |  |
| Mean blood pressure (continuous) |  | 1.00 [0.99, 1.00] | 0.342 | 1.00 [1.00, 1.01] | 0.330 | 1.00 [1.00, 1.00] | 0.742 |  |  |

* AMD: absent/missing data. aIRR: adjusted incidence risk ratio. 95% CI: 95% Confidence interval. MD: missing data. RDT: rapid diagnostic test. MFD: microfilarial density. mf/mL: microfilariae per milliliter of blood. epg: eggs per gram of stool.
