# Supplementary material for: Disability and quality of life assessment using WHODAS-12 items 2.0 and EQ-5D-5L in a rural area endemic for loiasis in the Republic of Congo: A population-based cross-sectional study (the MorLo project)
Source: PLoS Negl Trop Dis. 2025 Sep 15;19(9):e0013491. doi: 10.1371/journal.pntd.0013491 (PMC12449028; doi:10.1371/journal.pntd.0013491)
Supplement: S4 Table — (DOCX) [file pntd.0013491.s006.docx]

**S4 Table.** Disabilities for the 6 dimensions from the EQ-5D-5L questionnaire.

|  |  | EQmobility | | EQautonomy | | EQdaily | | EQpain | | EQanxiety | |
| --- | --- | --- | --- | --- | --- | --- | --- | --- | --- | --- | --- |
|  |  | Mean | SD | Mean | SD | Mean | SD | Mean | SD | Mean | SD |
| Total |  | 25.2 | 30.6 | 5.2 | 15.6 | 33.6 | 33.6 | 51.1 | 25.2 | 34.2 | 29.4 |
| Sex | Female | 36.0 | 31.2 | 6.7 | 17.9 | 43.8 | 32.4 | 55.8 | 23.1 | 40.7 | 28.9 |
|  | Male | 18.7 | 28.4 | 4.4 | 13.9 | 27.5 | 32.9 | 48.2 | 25.9 | 30.4 | 29.0 |
| Age (y.o.) | 18-28 | 7.8 | 19.7 | 0.6 | 3.8 | 15.7 | 25.2 | 39.5 | 27.2 | 24.1 | 23.8 |
|  | 29-38 | 16.6 | 26.7 | 4.7 | 14.4 | 29.7 | 31.9 | 45.1 | 26.4 | 29.4 | 28.6 |
|  | 39-48 | 19.1 | 28.4 | 5.5 | 15.9 | 26.2 | 32.5 | 49.1 | 26.0 | 30.4 | 30.7 |
|  | 49-58 | 22.7 | 29.9 | 4.2 | 12.9 | 30.4 | 32.0 | 49.6 | 25.6 | 33.4 | 30.0 |
|  | 59-68 | 33.2 | 30.3 | 6.4 | 17.1 | 41.9 | 34.0 | 57.4 | 20.0 | 38.8 | 28.3 |
|  | >68 | 49.2 | 30.1 | 9.1 | 22.1 | 55.7 | 31.8 | 61.9 | 21.4 | 47.2 | 27.5 |
| Eye worm episodes | 0 | 22.6 | 30.2 | 4.8 | 14.8 | 31.4 | 34.1 | 48.8 | 26.4 | 31.2 | 29.2 |
|  | 1-5 | 24.3 | 30.2 | 6.7 | 18.3 | 31.4 | 33.8 | 50.0 | 25.6 | 35.3 | 29.8 |
|  | 6-10 | 29.6 | 30.7 | 4.3 | 13.3 | 37.6 | 32.0 | 55.7 | 22.0 | 36.9 | 28.6 |
|  | >10 | 34.2 | 31.6 | 8.2 | 19.1 | 38.3 | 33.2 | 53.8 | 22.3 | 37.7 | 30.7 |
|  | AMD* | 24.9 | 30.8 | 4.5 | 14.2 | 35.9 | 33.6 | 52.6 | 24.6 | 36.8 | 29.3 |
| Calabar episodes | 0 | 24.9 | 30.2 | 5.3 | 15.6 | 32.8 | 33.8 | 50.3 | 25.4 | 33.2 | 29.6 |
|  | 1-5 | 24.8 | 29.7 | 5.6 | 16.7 | 33.0 | 33.6 | 48.7 | 24.6 | 34.8 | 28.1 |
|  | 6-10 | 26.7 | 33.2 | 5.1 | 14.7 | 34.8 | 32.1 | 53.4 | 25.9 | 34.6 | 29.8 |
|  | >10 | 26.6 | 31.5 | 6.9 | 19.3 | 33.0 | 35.0 | 56.4 | 22.4 | 36.2 | 30.8 |
|  | AMD | 25.3 | 31.1 | 4.5 | 14.2 | 36.3 | 33.7 | 52.6 | 24.9 | 36.7 | 29.2 |
| *Loa* MFD (mf/mL) | 0 | 26.1 | 31.0 | 5.4 | 15.5 | 33.2 | 33.8 | 51.0 | 25.2 | 34.4 | 29.7 |
|  | 1-7,999 | 24.1 | 29.7 | 5.1 | 16.4 | 35.9 | 34.0 | 51.2 | 24.6 | 33.4 | 28.5 |
|  | -19,999 | 23.1 | 31.0 | 5.8 | 15.8 | 26.5 | 30.9 | 51.2 | 27.0 | 35.8 | 28.6 |
|  | >19,999 | 21.1 | 29.2 | 3.1 | 8.4 | 36.7 | 32.4 | 50.8 | 26.6 | 34.4 | 32.8 |
| *Loa* RDT (Intensity)* | 0 | 28.4 | 31.6 | 4.7 | 12.7 | 38.6 | 36.1 | 53.8 | 22.7 | 37.3 | 29.9 |
|  | 1-2 | 27.9 | 31.3 | 6.9 | 17.6 | 38.0 | 34.2 | 51.5 | 25.3 | 35.3 | 29.1 |
|  | 3-4 | 26.0 | 30.3 | 5.8 | 16.0 | 34.6 | 33.8 | 50.9 | 24.9 | 34.3 | 29.8 |
|  | 5-6 | 23.5 | 30.3 | 3.6 | 13.5 | 31.6 | 33.1 | 49.6 | 25.7 | 32.6 | 28.9 |
|  | >6 | 24.4 | 32.6 | 11.4 | 23.8 | 30.7 | 31.8 | 53.4 | 27.8 | 38.1 | 33.4 |
| Eosinophilia (× 10^9^ cells/L) | ≤2 | 24.7 | 30.4 | 5.1 | 15.5 | 33.6 | 33.6 | 50.4 | 25.6 | 34.8 | 29.6 |
|  | >2 | 26.0 | 30.3 | 6.0 | 15.8 | 33.9 | 34.3 | 50.5 | 23.5 | 30.2 | 29.2 |
|  | AMD | 28.4 | 33.9 | 5.1 | 16.1 | 33.6 | 33.1 | 58.9 | 22.6 | 36.6 | 27.3 |
| Main occupation | Other | 25.2 | 32.3 | 5.7 | 16.8 | 32.4 | 35.9 | 49.4 | 26.5 | 34.8 | 30.3 |
|  | Farmer | 25.2 | 30.2 | 5.1 | 15.2 | 33.9 | 33.0 | 51.5 | 24.8 | 34.1 | 29.2 |
| Marital status | Couple | 22.1 | 29.3 | 4.1 | 13.6 | 29.0 | 33.4 | 48.6 | 25.5 | 30.3 | 28.8 |
|  | Single | 30.7 | 32.0 | 7.3 | 18.4 | 41.6 | 32.6 | 55.2 | 24.0 | 41.0 | 29.3 |
| SCD status | HbAA | 25.5 | 30.7 | 5.1 | 15.4 | 33.8 | 33.6 | 51.8 | 24.9 | 35.1 | 29.7 |
|  | HbAS | 24.1 | 30.2 | 5.7 | 15.7 | 32.5 | 33.6 | 48.0 | 26.0 | 31.0 | 28.3 |
| Tobacco use* | No | 26.6 | 30.9 | 5.3 | 15.6 | 34.4 | 33.8 | 51.5 | 25.1 | 34.3 | 29.4 |
|  | Yes | 19.4 | 28.7 | 5.2 | 15.8 | 30.8 | 33.4 | 49.5 | 25.3 | 34.3 | 29.9 |
|  | 0 | 23.9 | 29.7 | 4.6 | 14.4 | 31.4 | 33.2 | 50.1 | 25.4 | 31.4 | 28.7 |
| *Ascaris lumbricoides* (epg) | 1-1,000 | 24.8 | 30.5 | 5.8 | 16.5 | 33.4 | 33.4 | 50.6 | 24.8 | 35.2 | 30.6 |
|  | >1,000 | 31.6 | 33.0 | 8.1 | 19.4 | 38.0 | 35.4 | 53.9 | 25.0 | 38.0 | 30.5 |
|  | AMD | 24.7 | 31.0 | 4.5 | 14.4 | 35.7 | 33.5 | 51.8 | 25.1 | 36.9 | 28.8 |
| *Trichuris trichiura* infection | No | 25.4 | 30.4 | 5.2 | 15.4 | 33.1 | 33.9 | 50.5 | 25.1 | 31.6 | 29.5 |
|  | Yes | 25.1 | 30.8 | 6.2 | 17.1 | 32.7 | 33.2 | 51.6 | 25.5 | 38.6 | 29.1 |
|  | AMD | 24.7 | 31.1 | 4.6 | 14.5 | 35.8 | 33.6 | 51.9 | 25.2 | 36.9 | 28.9 |
|  |  |  |  |  |  |  |  |  |  |  |  |

* AMD: absent/missing data. Other variables: total (4 missing data), *Loa* RDT (18), Tobacco use (8), SCD status (7 invalids and 4 missing data). SD: standard deviation. MFD: microfilarial density. mf/mL: microfilariae per milliliter of blood. RDT: Rapid diagnostic test. SCD: sickle cell disease. epg: eggs per gram of stool
