# Supplementary material for: Disability and quality of life assessment using WHODAS-12 items 2.0 and EQ-5D-5L in a rural area endemic for loiasis in the Republic of Congo: A population-based cross-sectional study (the MorLo project)
Source: PLoS Negl Trop Dis. 2025 Sep 15;19(9):e0013491. doi: 10.1371/journal.pntd.0013491 (PMC12449028; doi:10.1371/journal.pntd.0013491)
Supplement: S5 Table — (DOCX) [file pntd.0013491.s007.docx]

**S5 Table.** Saturated multivariable analyses on EQ-5D-5L scores (EQmobility, EQautonomy, EQdaily, and EQpain).

|  |  | EQmobility |  | EQautonomy |  | EQdaily |  | EQpain |  |
| --- | --- | --- | --- | --- | --- | --- | --- | --- | --- |
| Variable |  | aIRR [95% CI] | p | aIRR [95% CI] | p | aIRR [95% CI] | p | aIRR [95% CI] | p |
| Eye worm episodes (Ref: 0) | 1-5 | 1.05 [0.84, 1.31] | 0.691 | 1.47 [0.80, 2.71] | 0.214 | 0.98 [0.82, 1.18] | 0.849 | 1.01 [0.89, 1.16] | 0.830 |
|  | 6-10 | 1.20 [0.95, 1.52] | 0.129 | 0.93 [0.46, 1.92] | 0.853 | 1.13 [0.93, 1.36] | 0.228 | 1.09 [0.95, 1.26] | 0.224 |
|  | >10 | 1.71 [1.27, 2.29] | <0.001 | 2.27 [1.00, 5.15] | 0.050 | 1.25 [0.97, 1.61] | 0.088 | 1.08 [0.89, 1.31] | 0.411 |
|  | AMD* | 0.80 [0.32, 2.00] | 0.640 | 1.04 [0.04, 29.78] | 0.983 | 0.79 [0.37, 1.66] | 0.531 | 1.06 [0.61, 1.82] | 0.843 |
| Calabar swelling episodes (Ref: 0) | 1-5 | 0.93 [0.73, 1.20] | 0.595 | 0.77 [0.38, 1.58] | 0.482 | 1.00 [0.81, 1.23] | 0.978 | 0.96 [0.82, 1.12] | 0.625 |
|  | 6-10 | 0.99 [0.75, 1.31] | 0.929 | 1.00 [0.44, 2.25] | 0.999 | 1.03 [0.82, 1.30] | 0.786 | 1.04 [0.88, 1.24] | 0.615 |
|  | >10 | 0.90 [0.60, 1.34] | 0.595 | 0.82 [0.29, 2.35] | 0.711 | 0.90 [0.64, 1.25] | 0.527 | 1.12 [0.89, 1.41] | 0.343 |
|  | AMD | 1.93 [0.83, 4.49] | 0.127 | 1.63 [0.06, 43.07] | 0.771 | 1.71 [0.85, 3.45] | 0.133 | 1.16 [0.69, 1.95] | 0.582 |
| *Loa* RDT (Intensity) (Ref: 0) | 1-2 | 1.05 [0.73, 1.51] | 0.781 | 1.78 [0.62, 5.07] | 0.283 | 1.07 [0.80, 1.43] | 0.664 | 1.01 [0.80, 1.27] | 0.936 |
|  | 3-4 | 0.96 [0.70, 1.30] | 0.781 | 1.32 [0.53, 3.26] | 0.547 | 0.95 [0.74, 1.22] | 0.695 | 0.98 [0.81, 1.19] | 0.819 |
|  | 5-6 | 0.99 [0.72, 1.36] | 0.967 | 0.91 [0.36, 2.32] | 0.848 | 0.92 [0.71, 1.18] | 0.501 | 1.00 [0.82, 1.22] | 0.993 |
|  | >6 | 1.10 [0.69, 1.76] | 0.679 | 2.88 [0.85, 9.74] | 0.088 | 0.93 [0.63, 1.36] | 0.696 | 1.10 [0.83, 1.45] | 0.497 |
| *Loa* MFD (mf/mL) (Ref: 0) | 1-7,999 | 0.94 [0.78, 1.12] | 0.474 | 0.83 [0.51, 1.35] | 0.442 | 1.07 [0.93, 1.24] | 0.325 | 1.01 [0.91, 1.12] | 0.855 |
|  | 8,000-19,999 | 0.88 [0.64, 1.22] | 0.446 | 1.06 [0.45, 2.49] | 0.895 | 0.80 [0.61, 1.06] | 0.123 | 0.99 [0.82, 1.20] | 0.945 |
|  | >19.999 | 0.86 [0.55, 1.35] | 0.505 | 0.73 [0.19, 2.74] | 0.642 | 1.18 [0.85, 1.65] | 0.320 | 1.02 [0.79, 1.32] | 0.864 |
| Sex (Ref: female) | Male | 0.66 [0.55, 0.79] | <0.001 | 0.99 [0.60, 1.65] | 0.973 | 0.83 [0.71, 0.96] | 0.012 | 0.97 [0.87, 1.08] | 0.566 |
| Age (Ref: 18-28 y.o.) | 29-38 | 1.96 [1.23, 3.13] | 0.005 | 7.15 [1.39, 36.70] | 0.018 | 1.81 [1.29, 2.54] | 0.001 | 1.24 [0.99, 1.55] | 0.066 |
|  | 39-48 | 2.09 [1.33, 3.28] | 0.001 | 7.92 [1.58, 39.59] | 0.012 | 1.59 [1.14, 2.21] | 0.006 | 1.29 [1.04, 1.61] | 0.020 |
|  | 49-58 | 2.57 [1.65, 4.00] | <0.001 | 8.31 [1.68, 41.18] | 0.010 | 1.80 [1.30, 2.49] | <0.001 | 1.34 [1.08, 1.66] | 0.007 |
|  | 59-68 | 3.12 [1.99, 4.89] | <0.001 | 10.19 [1.99, 52.09] | 0.005 | 2.08 [1.49, 2.91] | <0.001 | 1.47 [1.17, 1.83] | 0.001 |
|  | >68 | 4.29 [2.71, 6.78] | <0.001 | 11.20 [2.14, 58.69] | 0.004 | 2.46 [1.75, 3.46] | <0.001 | 1.51 [1.19, 1.90] | 0.001 |
| *Trichuris trichiura* infection (Ref: no) | Yes | 0.93 [0.75, 1.14] | 0.478 | 0.96 [0.53, 1.74] | 0.887 | 0.96 [0.81, 1.14] | 0.646 | 1.02 [0.90, 1.16] | 0.721 |
|  | AMD | 0.45 [0.09, 2.38] | 0.347 | 5.6 [0.00, 22.7] | 0.997 | 1.21 [0.26, 5.58] | 0.807 | 1.11 [0.35, 3.59] | 0.858 |
| *Ascaris lumbricoides* (epg) (Ref: 0) | 1-1,000 | 1.07 [0.88, 1.31] | 0.500 | 1.28 [0.73, 2.25] | 0.391 | 1.06 [0.90, 1.25] | 0.482 | 1.01 [0.89, 1.14] | 0.899 |
|  | >1,000 | 1.32 [1.03, 1.69] | 0.030 | 1.64 [0.81, 3.34] | 0.170 | 1.17 [0.95, 1.44] | 0.135 | 1.05 [0.90, 1.22] | 0.559 |
|  | AMD | 1.66 [0.33, 8.45] | 0.539 | 0.01 [0.00, 32.2] | 0.997 | 0.74 [0.16, 3.33] | 0.696 | 0.83 [0.26, 2.61] | 0.744 |
| Eosinophilia (× 10^9^ cells/L) (Ref. ≤2) | >2 | 1.12 [0.91, 1.39] | 0.297 | 1.09 [0.60, 1.97] | 0.770 | 1.02 [0.86, 1.22] | 0.809 | 1.00 [0.88, 1.13] | 0.964 |
|  | AMD | 1.17 [0.87, 1.58] | 0.291 | 1.23 [0.53, 2.83] | 0.631 | 1.05 [0.81, 1.34] | 0.720 | 1.16 [0.98, 1.39] | 0.089 |
| Sickle cell status (Ref: HbAA) | HbAS | 0.93 [0.77, 1.11] | 0.419 | 1.15 [0.69, 1.89] | 0.596 | 0.94 [0.81, 1.09] | 0.385 | 0.93 [0.83, 1.04] | 0.198 |
| Body mass index (continuous) |  | 1.01 [0.99, 1.03] | 0.417 | 1.07 [1.00, 1.14] | 0.069 | 1.00 [0.98, 1.02] | 0.990 | 1.00 [0.99, 1.02] | 0.726 |
| Main occupation (Ref: other) | Farmer | 0.89 [0.73, 1.09] | 0.259 | 0.74 [0.42, 1.29] | 0.283 | 0.95 [0.81, 1.12] | 0.573 | 0.97 [0.86, 1.10] | 0.636 |
| Marital status (Ref: as a couple) | Alone | 1.15 [0.97, 1.36] | 0.097 | 1.69 [1.07, 2.67] | 0.023 | 1.27 [1.11, 1.45] | <0.001 | 1.11 [1.00, 1.22] | 0.045 |
| Years of schooling (continuous) |  | 0.96 [0.94, 0.98] | <0.001 | 0.92 [0.87, 0.99] | 0.017 | 0.95 [0.94, 0.97] | <0.001 | 0.98 [0.97, 0.99] | 0.005 |
| Tobacco use (Ref: no) | Yes | 1.01 [0.81, 1.27] | 0.925 | 1.10 [0.60, 2.02] | 0.760 | 1.08 [0.91, 1.29] | 0.369 | 1.01 [0.89, 1.14] | 0.927 |
| Mean blood pressure (continuous) |  | 1.00 [0.99, 1.00] | 0.766 | 0.99 [0.98, 1.01] | 0.293 | 1.00 [1.00, 1.00] | 0.873 | 1.00 [1.00, 1.00] | 0.703 |
|  |  | | |  |  |  |  |  |  |

* AMD: absent/missing data. aIRR: adjusted incidence risk ratio. 95% CI: 95% Confidence interval. MD: missing data. RDT: rapid diagnostic test. MFD: microfilarial density. mf/mL: microfilariae per milliliter of blood. epg: eggs per gram of stool.
