# Supplementary material for: Disability and quality of life assessment using WHODAS-12 items 2.0 and EQ-5D-5L in a rural area endemic for loiasis in the Republic of Congo: A population-based cross-sectional study (the MorLo project)
Source: PLoS Negl Trop Dis. 2025 Sep 15;19(9):e0013491. doi: 10.1371/journal.pntd.0013491 (PMC12449028; doi:10.1371/journal.pntd.0013491)
Supplement: S6 Table — (DOCX) [file pntd.0013491.s008.docx]

**S6 Table.** Saturated multivariable analyses on EQ-5D-5L scores (EQvas, EQvts, and EQanxiety).

|  |  | EQvas* |  | EQvts |  | EQanxiety |  |
| --- | --- | --- | --- | --- | --- | --- | --- |
| Variable |  | aIRR [95% CI] | p | ß-coefficients [95% CI] | p | aIRR [95% CI] | p |
| Eye worm episodes (Ref: 0) | 1-5 | 1.00 [0.95, 1.05] | 0.955 | -0.02 [-0.11, 0.06] | 0.554 | 1.09 [0.92, 1.29] | 0.303 |
|  | 6-10 | 0.97 [0.91, 1.02] | 0.223 | -0.04 [-0.13, 0.05] | 0.387 | 1.12 [0.94, 1.33] | 0.210 |
|  | >10 | 0.93 [0.86, 1.00] | 0.056 | -0.11 [-0.23, 0.01] | 0.063 | 1.19 [0.94, 1.50] | 0.140 |
|  | AMD* | 0.97 [0.78, 1.21] | 0.794 | 0.09 [-0.25, 0.43] | 0.610 | 0.99 [0.51, 1.92] | 0.978 |
| Calabar swelling episodes (Ref: 0) | 1-5 | 1.00 [0.95, 1.06] | 0.942 | 0.03 [-0.06, 0.13] | 0.479 | 0.98 [0.82, 1.18] | 0.844 |
|  | 6-10 | 1.01 [0.95, 1.08] | 0.726 | -0.04 [-0.14, 0.07] | 0.481 | 1.02 [0.82, 1.25] | 0.885 |
|  | >10 | 0.99 [0.91, 1.09] | 0.898 | -0.05 [-0.19, 0.10] | 0.504 | 1.07 [0.80, 1.42] | 0.653 |
|  | AMD | 1.02 [0.82, 1.27] | 0.847 | -0.24 [-0.56, 0.09] | 0.160 | 1.21 [0.64, 2.28] | 0.548 |
| *Loa* RDT (Intensity) (Ref: 0) | 1-2 | 1.03 [0.94, 1.13] | 0.491 | -0.04 [-0.18, 0.10] | 0.575 | 1.03 [0.78, 1.36] | 0.823 |
|  | 3-4 | 1.02 [0.94, 1.10] | 0.701 | 0.01 [-0.11, 0.13] | 0.828 | 0.95 [0.76, 1.20] | 0.693 |
|  | 5-6 | 1.02 [0.95, 1.11] | 0.570 | -0.01 [-0.13, 0.12] | 0.936 | 0.97 [0.76, 1.23] | 0.790 |
|  | >6 | 0.99 [0.88, 1.10] | 0.804 | -0.10 [-0.28, 0.07] | 0.242 | 1.17 [0.84, 1.62] | 0.358 |
| *Loa* MFD (mf/mL) (Ref: 0) | 1-7,999 | 0.98 [0.94, 1.02] | 0.291 | 0.00 [-0.06, 0.07] | 0.991 | 0.97 [0.85, 1.11] | 0.675 |
|  | 8,000-19,999 | 1.04 [0.96, 1.12] | 0.329 | 0.01 [-0.10, 0.13] | 0.823 | 1.03 [0.82, 1.29] | 0.819 |
|  | >19,999 | 0.99 [0.90, 1.09] | 0.847 | -0.01 [-0.17, 0.14] | 0.880 | 1.04 [0.77, 1.42] | 0.782 |
| Sex (Ref: female) | Male | 1.05 [1.01, 1.10] | 0.019 | 0.08 [0.01, 0.14] | 0.025 | 0.90 [0.78, 1.03] | 0.121 |
| Age (Ref: 18-28 y.o.) | 29-38 | 0.94 [0.87, 1.01] | 0.112 | -0.17 [-0.30, -0.05] | 0.006 | 1.30 [0.98, 1.73] | 0.069 |
|  | 39-48 | 0.94 [0.87, 1.02] | 0.119 | -0.20 [-0.32, -0.08] | 0.001 | 1.30 [0.98, 1.71] | 0.065 |
|  | 49-58 | 0.89 [0.83, 0.96] | 0.003 | -0.25 [-0.37, -0.13] | <0.001 | 1.47 [1.12, 1.92] | 0.006 |
|  | 59-68 | 0.83 [0.77, 0.90] | <0.001 | -0.30 [-0.43, -0.18] | <0.001 | 1.53 [1.15, 2.03] | 0.003 |
|  | >68 | 0.77 [0.71, 0.84] | <0.001 | -0.44 [-0.58, -0.31] | <0.001 | 1.64 [1.23, 2.19] | 0.001 |
| *Trichuris trichiura* infection (Ref: no) | Yes | 0.95 [0.91, 1.00] | 0.058 | -0.02 [-0.10, 0.06] | 0.609 | 1.20 [1.03, 1.39] | 0.021 |
|  | AMD | 0.85 [0.58, 1.25] | 0.417 | -0.14 [-0.76, 0.48] | 0.655 | 1.01 [0.31, 3.32] | 0.982 |
| *Ascaris lumbricoides* (epg) (Ref: 0) | 1-1,000 | 0.98 [0.93, 1.03] | 0.387 | -0.03 [-0.10, 0.05] | 0.452 | 1.10 [0.95, 1.27] | 0.222 |
|  | >1,000 | 0.97 [0.91, 1.03] | 0.266 | -0.08 [-0.18, 0.01] | 0.096 | 1.09 [0.91, 1.32] | 0.348 |
|  | AMD | 1.13 [0.77, 1.65] | 0.524 | 0.19 [-0.42, 0.80] | 0.541 | 1.06 [0.33, 3.38] | 0.925 |
| Eosinophilia (× 10^9^ cells/L) (Ref. ≤2) | >2 | 1.01 [0.96, 1.06] | 0.750 | 0.01 [-0.06, 0.09] | 0.726 | 0.86 [0.72, 1.01] | 0.067 |
|  | AMD | 0.90 [0.84, 0.97] | 0.004 | -0.12 [-0.23, -0.00] | 0.045 | 1.06 [0.85, 1.32] | 0.621 |
| Sickle cell disease (Ref: HbAA) | HbAS | 1.02 [0.98, 1.06] | 0.354 | 0.06 [-0.00, 0.13] | 0.056 | 0.90 [0.79, 1.04] | 0.146 |
| Body mass index (continuous) |  | 1.00 [0.99, 1.00] | 0.576 | -0.00 [-0.01, 0.01] | 0.379 | 1.01 [1.00, 1.03] | 0.154 |
| Main occupation (Ref: other) | Farmer | 1.05 [1.01, 1.10] | 0.029 | 0.06 [-0.02, 0.13] | 0.122 | 0.89 [0.77, 1.03] | 0.118 |
| Marital status (Ref: as a couple) | Alone | 0.92 [0.88, 0.95] | <0.001 | -0.13 [-0.19, -0.06] | <0.001 | 1.24 [1.10, 1.40] | 0.001 |
| Years of schooling (continuous) |  | 1.01 [1.01, 1.02] | <0.001 | 0.02 [0.02, 0.03] | <0.001 | 0.96 [0.95, 0.98] | <0.001 |
| Tobacco use (Ref: no) | Yes | 1.00 [0.95, 1.05] | 0.973 | -0.02 [-0.10, 0.06] | 0.638 | 1.12 [0.96, 1.31] | 0.139 |
| Mean blood pressure (continuous) |  | 1.00 [1.00, 1.00] | 0.376 | 0.00 [-0.00, 0.00] | 0.870 | 1.00 [1.00, 1.00] | 0.939 |
|  |  | | |  |  |  |  |

* AMD: absent/missing data. aIRR: adjusted incidence risk ratio. 95% CI: 95% Confidence interval. MD: missing data. RDT: rapid diagnostic test. MFD: microfilarial density. mf/mL: microfilariae per milliliter of blood. epg: eggs per gram of stool.
