# Supplementary material for: Disability and quality of life assessment using WHODAS-12 items 2.0 and EQ-5D-5L in a rural area endemic for loiasis in the Republic of Congo: A population-based cross-sectional study (the MorLo project)
Source: PLoS Negl Trop Dis. 2025 Sep 15;19(9):e0013491. doi: 10.1371/journal.pntd.0013491 (PMC12449028; doi:10.1371/journal.pntd.0013491)
Supplement: S1 Fig — (DOCX) [file pntd.0013491.s009.docx]

**S1 Fig.** Relationship between scores and number of years of schooling.

1. WHODAS domains.

Smoothed curve obtained using Stata’s LOWESS function. Blue = overall score; red = cognitive score; green = mobility score; yellow = selfcare score; violet = household score; and orange = participation score.

1. Number of days of disability from WHODAS questionnaire.

Smoothed curve obtained using Stata’s LOWESS function. Blue = mild disability; red = full disability; green = partial disability.

1. EQ-5D-5L dimensions.

Smoothed curve obtained using Stata’s LOWESS function. Blue = mobility score; red = autonomy score; green = daily score; yellow = pain score; and purple = anxiety score.
