# Supplementary figures and images for: Disability and quality of life assessment using WHODAS-12 items 2.0 and EQ-5D-5L in a rural area endemic for loiasis in the Republic of Congo: A population-based cross-sectional study (the MorLo project)
Source: PLoS Negl Trop Dis. 2025 Sep 15;19(9):e0013491. doi: 10.1371/journal.pntd.0013491 (PMC12449028; doi:10.1371/journal.pntd.0013491)

**S2 Fig.** Forest plot of the final models for WHODAS 2.0 questionnaire.


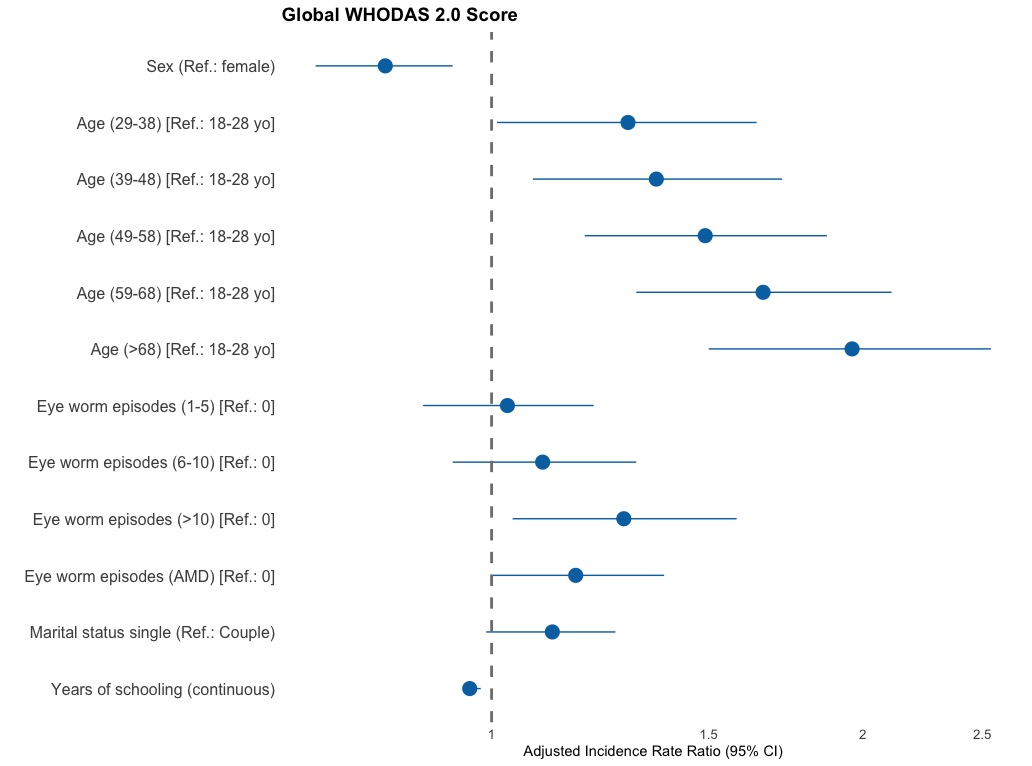


**
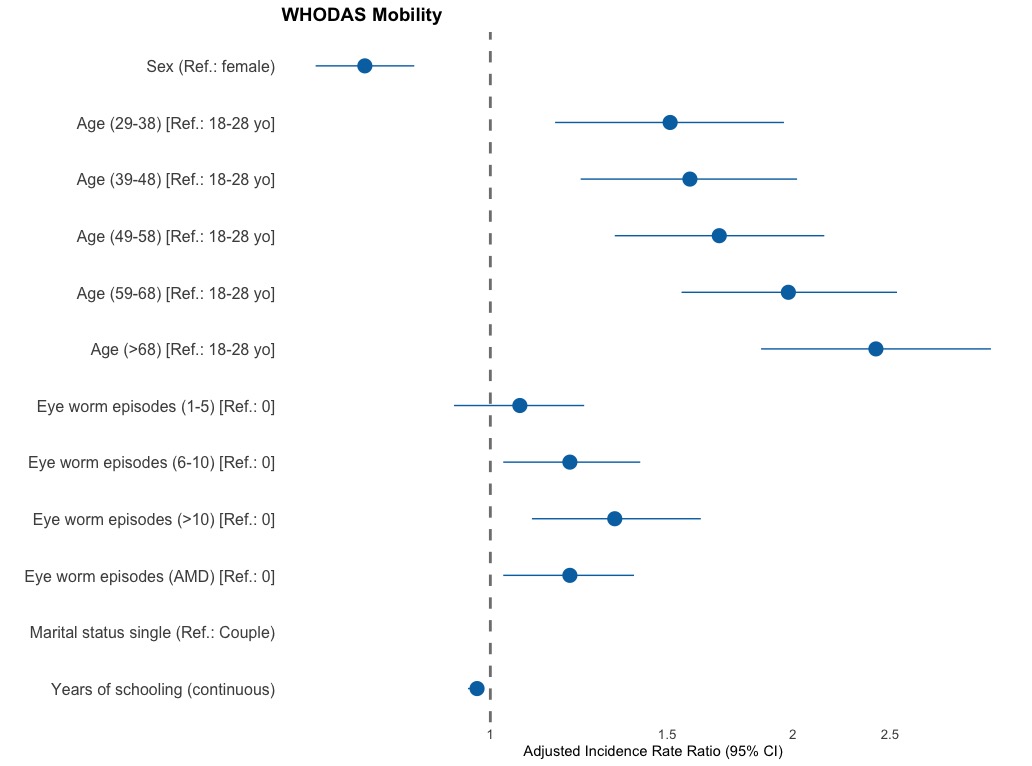
**

**
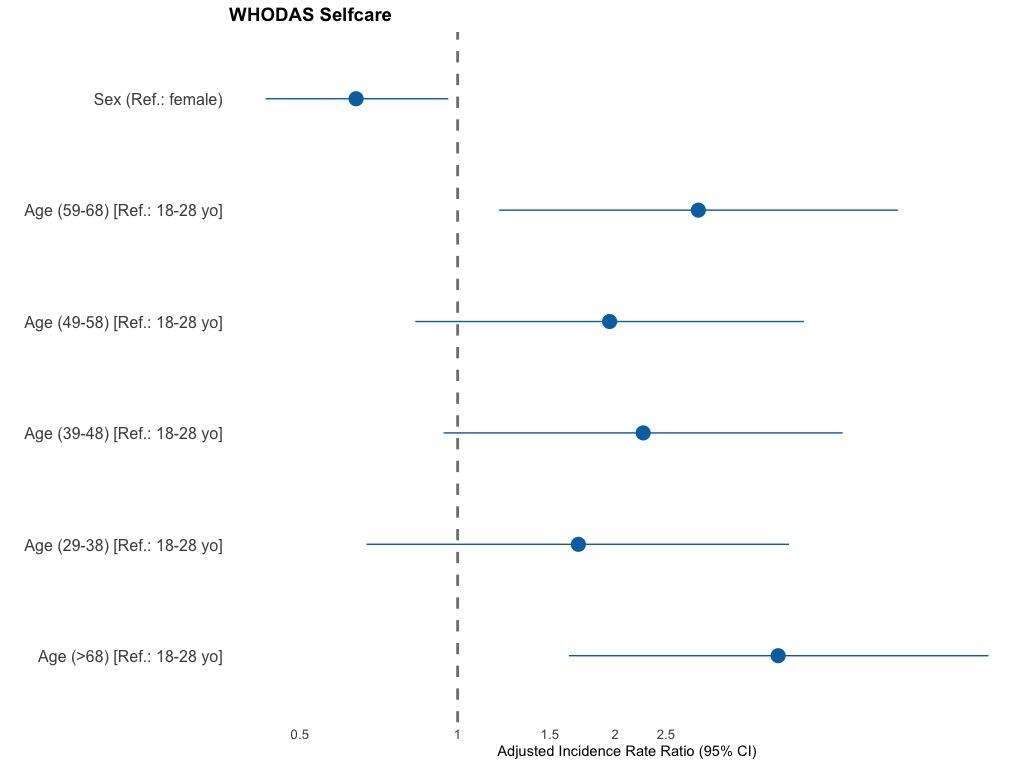
**

**
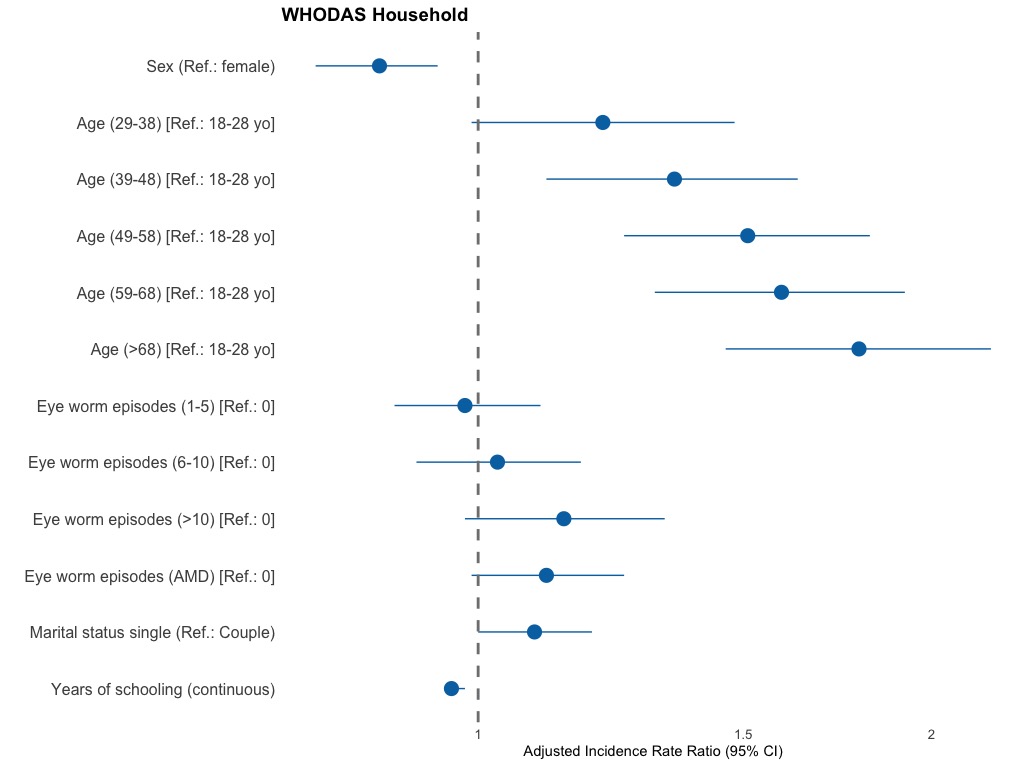
**

**
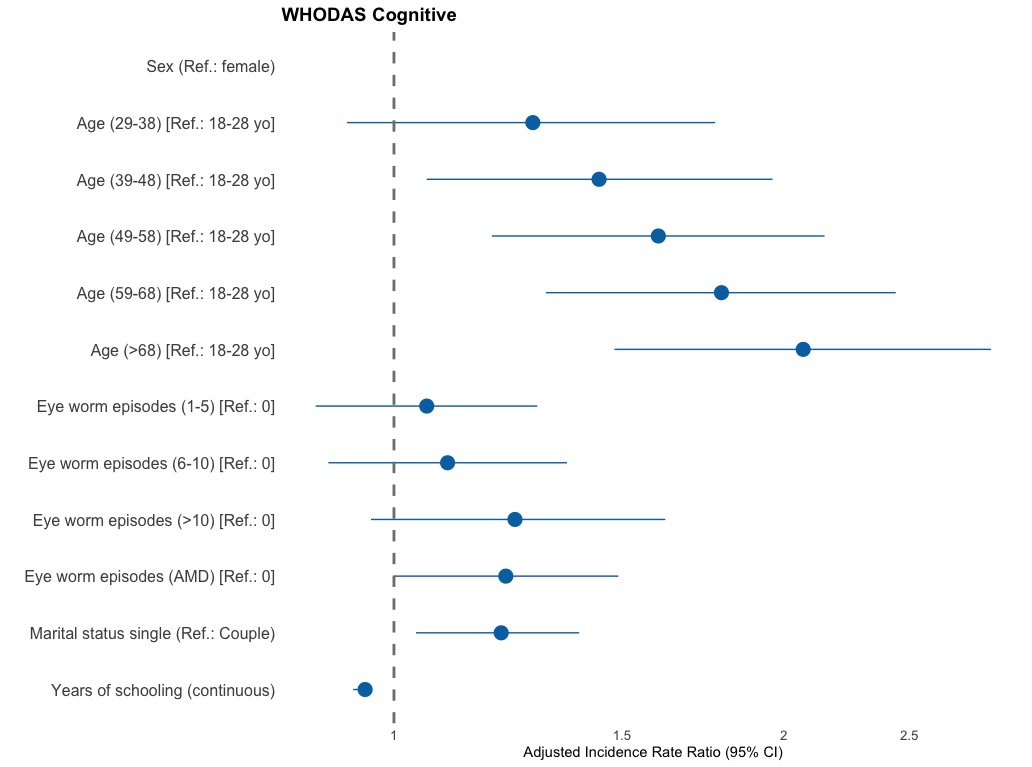
**

**
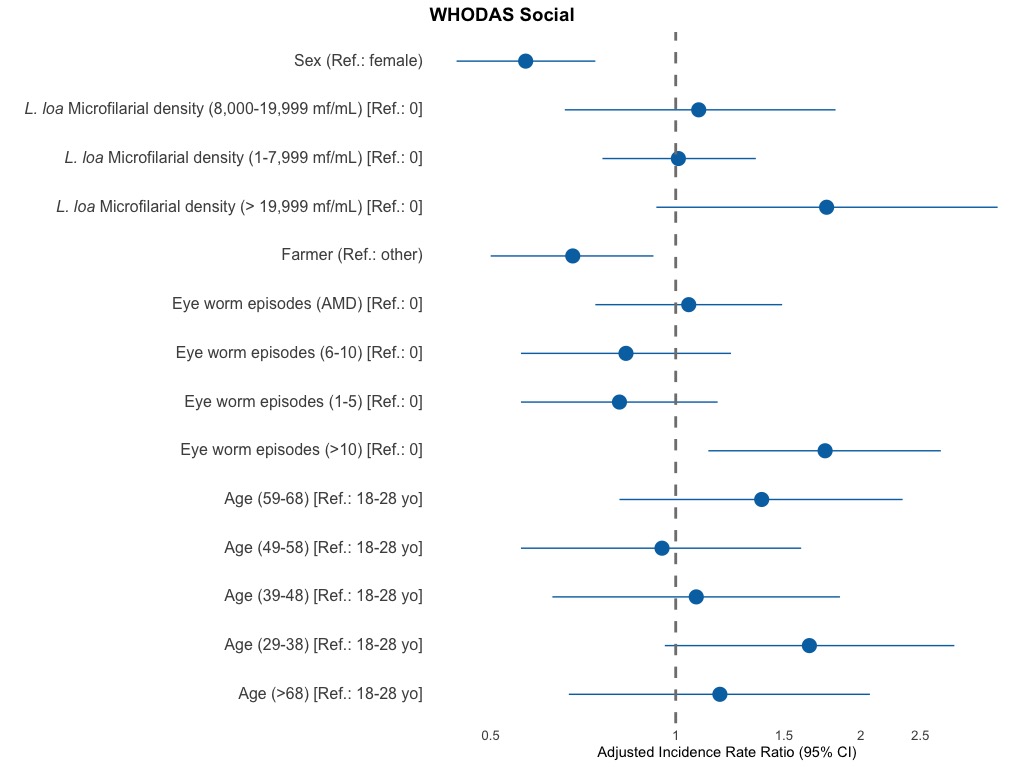
**

**
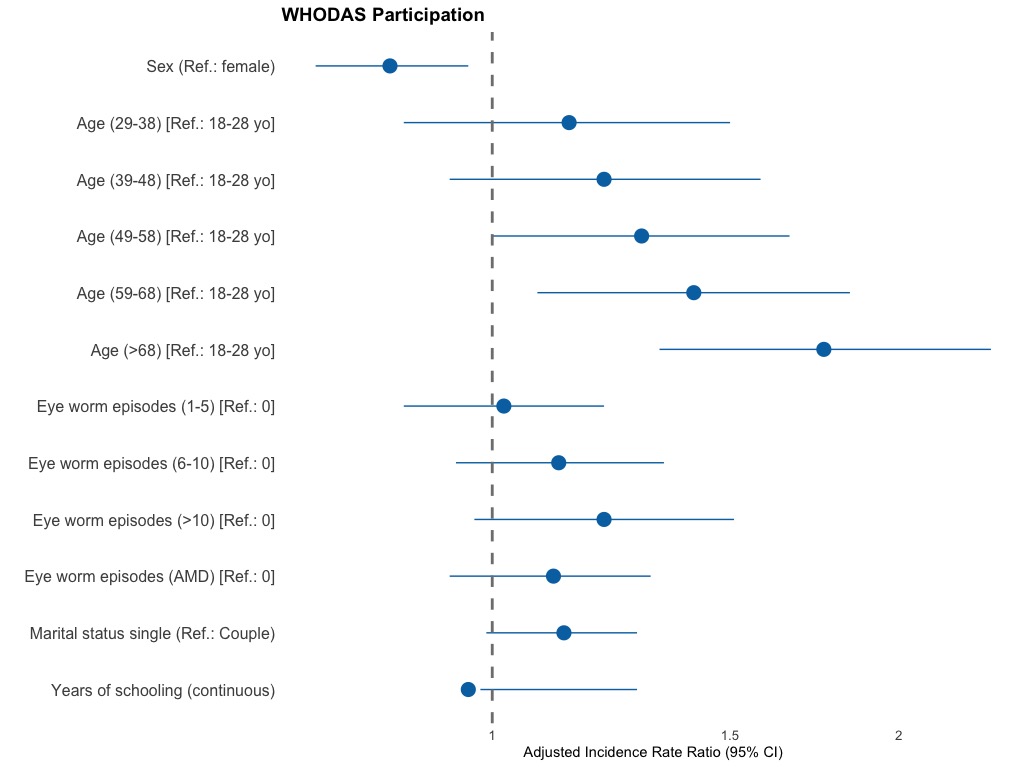
**

Supplement: S2 Fig — (DOCX) [file pntd.0013491.s010.docx]

**S3 Fig.** Forest plot of the final models for EQ-5D-5L questionnaire.

**
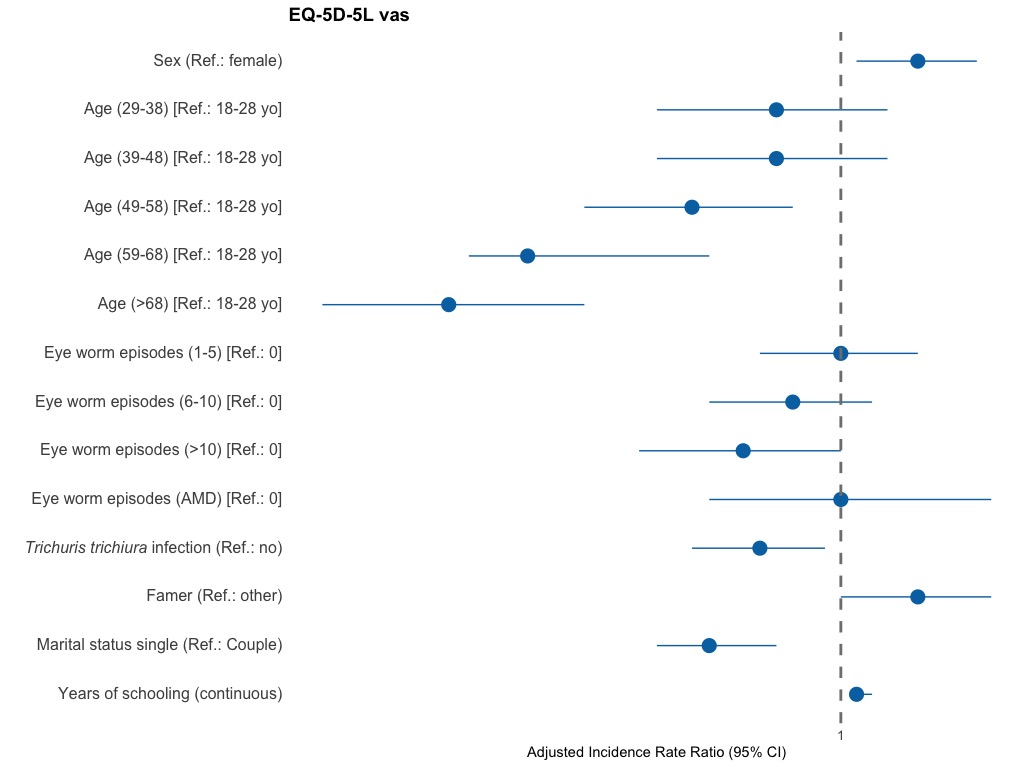
**

**
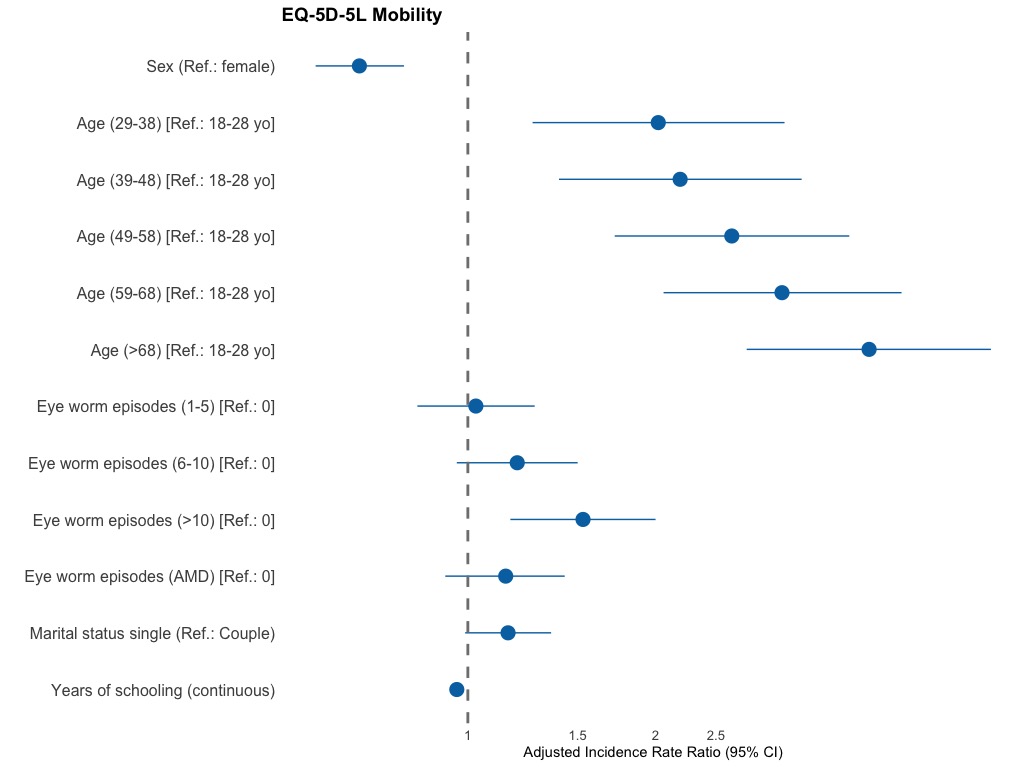
**

**
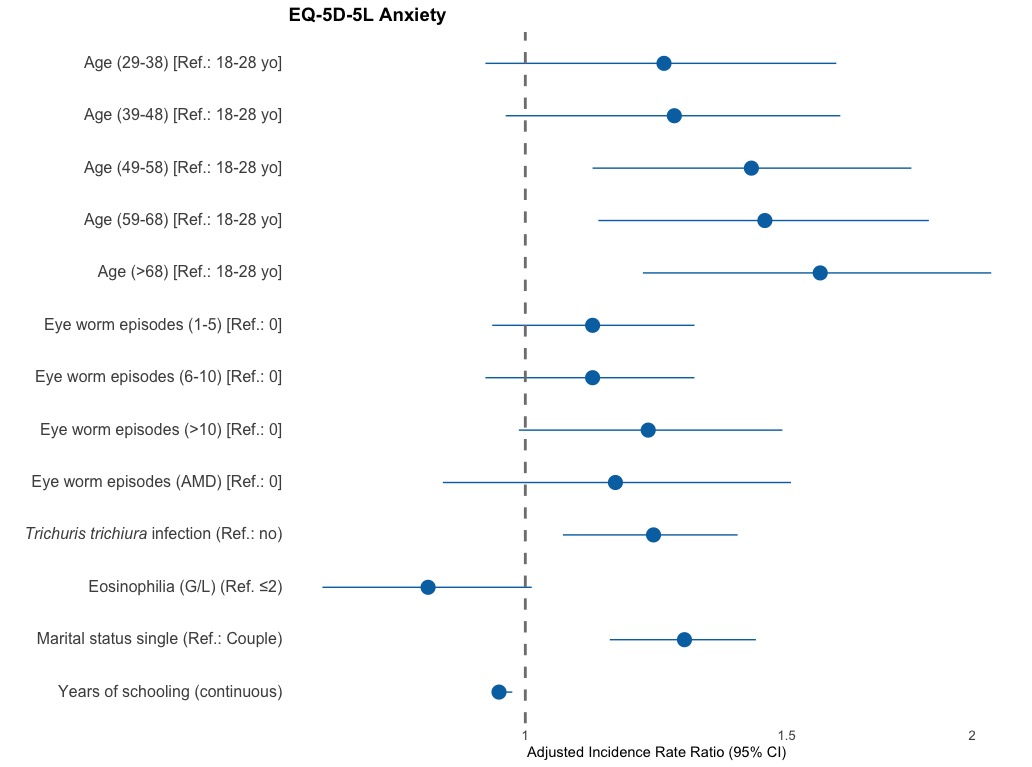
**

**
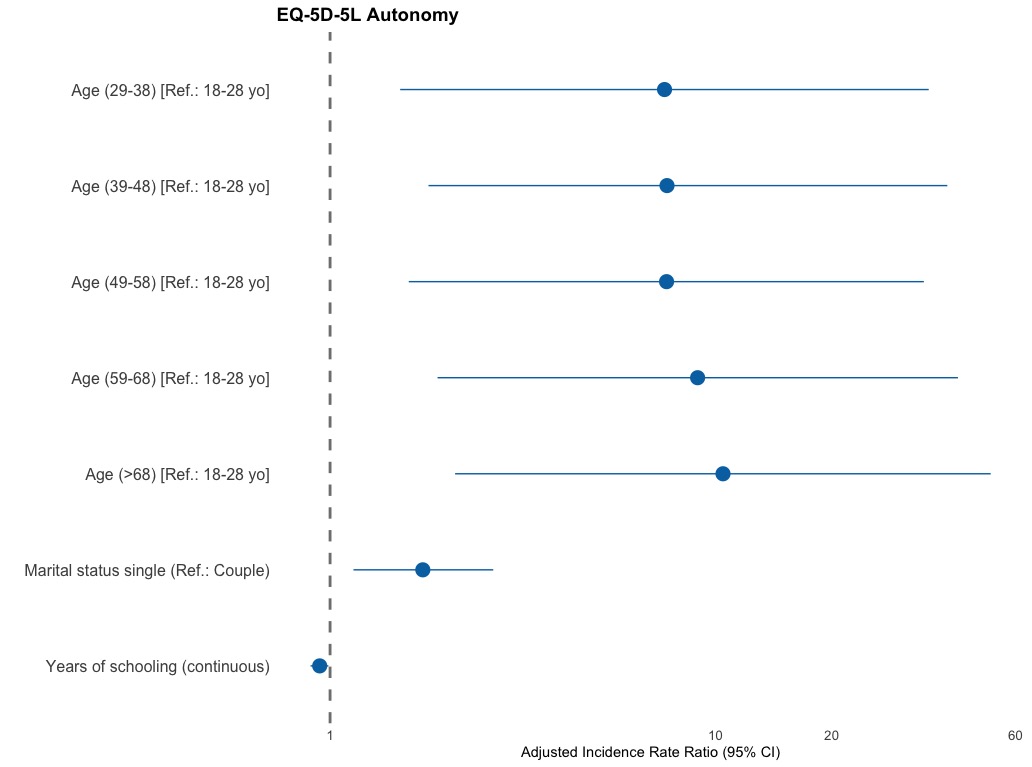
**


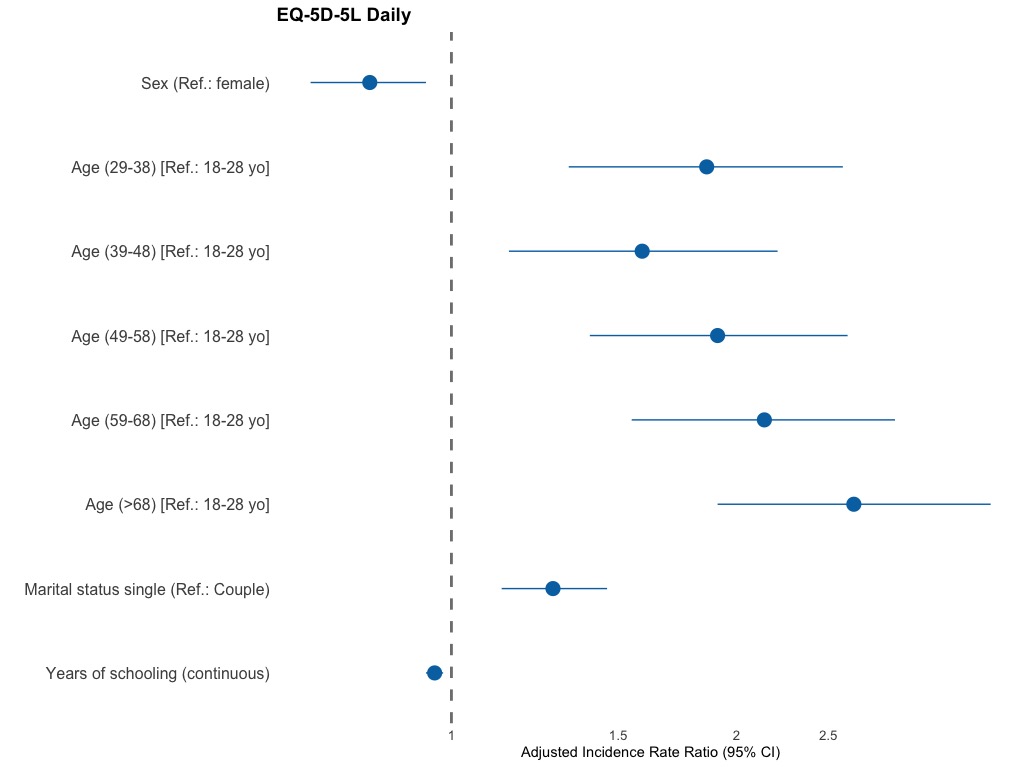


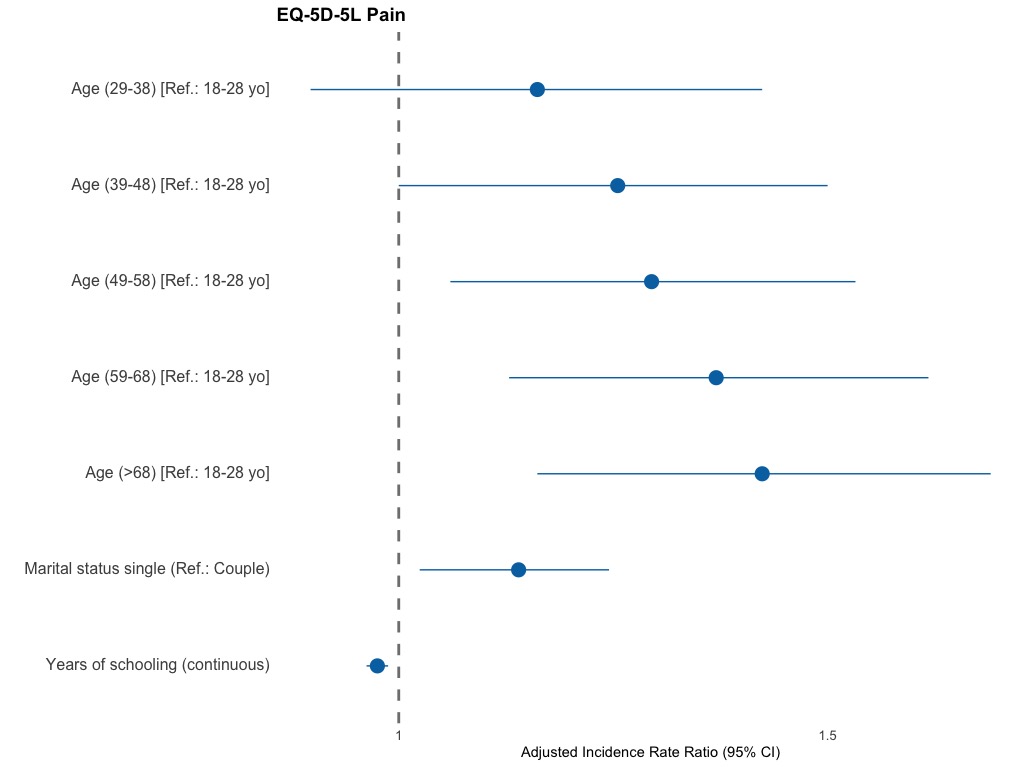

Supplement: S3 Fig — (DOCX) [file pntd.0013491.s011.docx]
