# Supplementary material for: Disability and quality of life assessment using WHODAS-12 items 2.0 and EQ-5D-5L in a rural area endemic for loiasis in the Republic of Congo: A population-based cross-sectional study (the MorLo project)
Source: PLoS Negl Trop Dis. 2025 Sep 15;19(9):e0013491. doi: 10.1371/journal.pntd.0013491 (PMC12449028; doi:10.1371/journal.pntd.0013491)
Supplement: Attachment — Submitted filename: PNTD 01363 COMMENTS.pdf [file pntd.0013491.s012.pdf]

PNTD D-24-01363 COMMENTS

This is an important study of one of a most common parasitic disease in Central Africa. Study on quality of life as well as disability in case of loiasis had not yet been carried out.

Although the thematic is of importance; major issues in study design, as well as in the analysis of the data, require major revisions before its consideration for publication.

Methods

Although information on the study villages is presented elsewhere, it is important to specify the known endemicity level of loiasis in order to have an idea of the exposure of the populations.

Moreover, although they are rural populations, their main income-generating activities are not specified, this can have an influence on the responses to the various questionnaires used.

L92: how was estimate of Fatigue measured.

L125-126: Deworming campaigns target children not included in the study population.

The authors should better justify the relevance of these campaigns in the design of the study. In addition, information on the molecules used for chemoprevention shall be provided.

The tests used for loiasis detection are also of concern

L132: why did the direct examination of 10µL of blood not examined?

What is the sensitivity of the 50µL Giemsa stained Thick smear?

What is the sensivity and specicity of the Loa loa RDT. Cross-reactivities have always been described? How were the participants without microfilaremia with a positive RDT?

Why are HbAS or HbSS patients individualized for data analysis. Why are other subpopulations such as malnourished people, those in particular occupations not?

L115-162: the authors have used only Kato-katz for the detection of intestinal parasites. This technique does not make it possible to properly detect all the parasitic stages of the protozoa, which themselves are likely to be responsible for nonspecific but suggestive symptoms of loiasis and responsible for disability

L168: please explain why "Lifetime"

L164-170: Explain how the symptoms were assessed? Calabar swellings are shown to do not be inflammatory, neither painful (unless extended) and to last several days.

The study population with microfilariae most likely has a chronic infection. the majority of these patients describe passages of a worm in the eye. A patient may have several episodes per day within a week. The authors should further describe the modalities of clinical evaluation and number of episodes throughout the year assessment and stratification.

Who administered the questionnaires, how was it administered so that ALL the participants, could understand the illustrations and questions.

Were there any awareness sessions or information during which loiasis symptoms were described, prior to the investigation?

There is also an ethical issue: there is no indication of participants treatment during the one year follow-up. Does it mean that infected participants as well as the symptomatic ones remained untreated during one year? What was the ethics committee recommendation regarding these aspects?

Results

L282-287: a study flow chart would be more appropriate as only 771 out of the 990 or 987 participants were tested for intestinal parasite carriage

What is the significance of analyzing the STH carriers when the main symptoms used to assess disability and quality of life are specific for loiasis.

On the other hand, associated symptoms such as generalized or localized pruritus, photophobia, are most likely to be associated with disability if chronic. These are the most frequent reasons for patient consultation. Pruritus is also a symptom described during certain STH and pathogenic c intestinal protozoa carriage. The fact that half of the participants tested carry at least one STH and that the protozoa have not been tested, should be better discussed.

Patients should be stratified according to the type of parasitism, and data from the questionnaire presented accordingly; i.e. absence of parasite, single parasite carriage, multiple parasite carriage (several microFilariae, Loa loa +STH).

Discussion

The discussion should be amended after considering all the comments above.

MINOR COMMENTS

L278: test for age distribution and choose mean or median, there is no need to present both

L287-290: the global prevalence of STH carriers should be presented. 50.9% of the tested participants had an STH infection, this is higher than Loiasis prevalence

L445: which symptom was painful?
