# Supplementary material for: Disability and quality of life assessment using WHODAS-12 items 2.0 and EQ-5D-5L in a rural area endemic for loiasis in the Republic of Congo: A population-based cross-sectional study (the MorLo project)
Source: PLoS Negl Trop Dis. 2025 Sep 15;19(9):e0013491. doi: 10.1371/journal.pntd.0013491 (PMC12449028; doi:10.1371/journal.pntd.0013491)
Supplement: Attachment — Submitted filename: PNTD-D-24-01363_R1_Reviewer 3.pdf [file pntd.0013491.s014.pdf]

Reviewer 3

PNTD-D-24-01363R1. Disability and quality of life assessment using WHODAS-12 items 2.0 and EQ-5D-5L in a rural area endemic for loiasis in the Republic of Congo: a population-based cross-sectional study (the MorLo project)

This paper, submitted in 2024, has already been refereed by two competent reviewers and the authors have prepared a point-by-point reply to these referees. I am not an expert in Quality of Life assessments, but the paper is important, and I read it with interest. The authors prepared a revised version that addressed the comments by the previous reviewers, so I do not have further comments on the Objectives, Methods, Results and Conclusions. However, some text can be improved for clarity and conciseness, as well as for grammar and punctuation. In the below, I list my comments by section of the paper, and by line. Figures and figure captions need some attention, as well as the supplementary materials (which I suggest be consolidated into a single S1 File, and referred to by (capital) letters rather than by numbers, following PLoS Negl Trop Dis guidelines when preparing a single Supporting Information file. I give some explanation as to how this can be done. The authors would need to change the way they refer to Supporting Information items in the Main Text, which in any case also requires some attention, as it is currently inconsistent.

My comments are as follows:

Abstract

Line 39: Where it says 'Calabar swellings episodes' it should say 'Calabar swelling episodes' (as adjectives should be singular'.

Author Summary

Line 64: Where it says 'Conversely', change to 'By contrast'.

Introduction

Line 78: Where it says '*Chrysops* sp.' It should say '*Chrysops* spp.' (as the authors are referring to more than one species).

Line 78: Where it says 'from southeast Benin …', the authors should provide a reference after 'Angola', as the paper by Zouré et al. does not include Benin, and RAPLOA surveys may not have been conducted there. Please cite Boussinesq & Gardon (1997) Prevalences of *Loa loa* microfilaraemia throughout the area endemic for the infection, published in Ann Trop Med Parasitol.

Line 81: Delete the determinate article 'the' before subconjunctival so it reads: 'such as pruritus, subconjunctival migration of the adult worms ("eye worm"), …'.

Lines 82-83: Change the semicolon to a comma and 'would be' to 'is', and add 'medical' before 'consultation', so the sentence reads: ', 'and is a frequent reason for medical consultation'. Would be is tentative, but as you are citing papers, this is not your conjecture.

Lines 83-84: Change ‘could have a significant public health impact’ to ‘has been reported to have a considerable public health impact’. Again, ‘could have’ is speculative, but then you are citing a paper quantifying the disease burden in Gabon. Also, reserve the use of ‘significant’ for ‘statistically significant’ throughout the paper to avoid any confusion.

Lines 84-85: add ‘of disease’ to ‘burden’ (because in other contexts burden is also used for worm burden, and you are specifically referring to the disease burden here). I suggest to re-write the sentence as follows: ‘Based on these symptoms alone, loiasis has been reported to have a considerable public health impact, with its estimated burden of disease in Gabon being close to that of schistosomiasis.’

Line 88: Add ‘progeny’ to larval’ (because there are other larval stages in the life-cycle (for instance within an infected/infective vector), so it reads: ‘larval progeny stages of the parasite’.

Line 91: Change ‘impact’ to ‘detrimentally affect’ so it reads ‘some manifestations might detrimentally affect everyday activity’. In this way you also avoid the overuse of the word ‘impact’.

Line 94: Change ‘microfilarial density (MFD) is linked to a globally impaired QoL’ to ‘microfilarial density (MFD) is linked to an overall impaired QoL’. At this stage you have not yet introduced the global score terminology.

Lines 95-97: The word ‘Conversely’ means ‘the opposite’, which I don’t think conveys the message well. I suggest to change to ‘Furthermore’. Also change ‘potential impact’ to ‘potential effect’ so it reads: ‘Furthermore, as no study to date has demonstrated an association between adult worms and severe complications, it is difficult to extrapolate their potential effect on QoL.’

Lines 100-101: I don’t think that starting the sentence with ‘We hypothesize that …’ is necessary, because you are using ‘could contribute’ as a conjecture. Also, ‘assuming a certain chronicity of these symptoms’ could be omitted (as you are already mentioning ‘chronic pain or mobility’.
I suggest to simplify this sentence to read: ‘Adult worms, due to their migration, could contribute to symptoms related to chronic pain or mobility disorders’ for clarity and conciseness.

Study design

Line 129: Add ‘the’ to ‘baseline’, because you are referring to a particular cohort, so it reads: ‘It was conducted at the baseline of the cohort from May 16 to June 11, 2022’.

Line 131: Add ‘of the Republic of Congo’ to ‘Lékoumou division’, so it reads: ‘Lékoumou division, of the Republic of Congo’.

Line 132: Delete the full stop after 20 so it reads: ‘prevalence of 20%’. Add ‘and’ and change ‘hypo-meso endemic’ to ‘hypo- to mesoendemic’ so it reads: ‘and hypo-to mesoendemic for genito-urinary schistosomiasis’.

Line 135: Delete ‘the’ before ‘Congo’. Nowhere else in the paper this is used, so it reads: ‘Republic of Congo’.

Line 137: The text mentions a previous clinical trial, but no reference is given. Please give a reference here to a published paper or to the registration of this clinical trial.

Laboratory procedures

Line 158: Add ‘The’ before ‘incubation medium’, so it reads: ‘The incubation medium was examined’.

Line 163: Change ‘Last’ to ‘Also,’ so it reads: ‘Also, we performed a urinalysis’. You have a ‘Finally’ later (in Line 168), so this cannot be ‘Last’.

Line 169: You have not yet defined STH. Please change to: ‘stool samples for soil-transmitted helminthiasis (STH) and intestinal schistosomiasis screening’.

Disability and quality of life assessment

Line 204: Change ‘First’ to ‘Firstly’, as the former is used in informal writing and speech, while ‘firstly’ is more formal.

Line 214: Give a reference for the "eq5d" R package, as follows: Morton F, Nijjar JS. Calculating EQ-5D indices and summarising profiles with eq5d. 2025. Available from: https://cran.r-project.org/web/packages/eq5d/vignettes/eq5d.html.

Power calculation

Line 231: The reasoning behind ‘The sample size for the underlying cohort study had been calculated (991) to show an increase in the risk of malaria and pneumonia of x1.4 and x2.4 for microfilaremic vs amicrofilaremic individuals’ is not entirely clear. Please clarify why x1.4 and x2.4 were used.

Line 236: A global WHODAS score >25 is referred to as indicating ‘moderate disability’. But later on (Line 279), it is said that a score >50 is indicative of ‘severe global disability’. Should this moderate disability be >25 to 50? I am not a WHODAS expert, but other readers may not be either, so worth clarifying?

Line 256: Add reference to the "psych" package in R, as follows: Revelle W. psych: procedures for psychological, psychometric, and personality research. 2025. Available from: https://cran.r-project.org/web/packages/psych/index.html.

Disability assessment

Line 277: Change ‘variable’ to ‘variable(s)’.

Line 278: Change ‘significant’ to ‘statistically significant’.

Line 278: Change ‘Last’ to ‘Finally’.

Lines 279-280: Where it says: ‘…score >25 (moderate global disability – or significant disability) and >50 (severe global disability)’, is there an intermediate value missing for significant disability? Is a score >25 to 50 indicative of moderate disability or significant disability? See also question in Line 236 above. I was confused about this and other readers may be too. Worth clarifying if possible?

Line 283: Change ‘variable’ to ‘variable(s)’.

Line 287: ‘Given that we followed the instructions to analyze these questionnaires’, which instructions are these precisely? Not very clear.

Line 288: '… significant number of scores evaluated'. Do you mean considerable? Statistically significant Please clarify.

Line 299: Delete 'last' so it reads 'and a three-way interaction term …'.

Description of the study population

Line 309: Change 'year of schooling' to 'years of schooling'.

Line 315: Change 'sp.' to 'spp.'

Line 317: Change 'positive at' to 'positive for'.

Line 324: Delete 'Conversely' so it reads 'They did not differ in terms of sex ratio …'.

Line 325: Delete 'Last' so it reads: 'Regarding the STH group …'.

Line 329: Delete 'Conversely' so it reads 'They did not differ in terms of sex ratio …'.

Internal consistency of the questionnaires

Lines 333-335: Change 'with a total omega coefficient of 0.9, a correlation of scores with a factor of 0.97 and a multiple R square of scores with a factor of 0.94' to 'with a total omega coefficient equal to 0.9, a correlation of scores with a factor equal to 0.97, and a multiple R square of scores with a factor equal to 0.94'. I think this makes the meaning clearer.

Correlations, multicollinearity, and interaction terms

Line 342: Since you are starting a new paragraph, write *Trichuris* in full.

Line 348: Clarify if you mean statistically significant correlations.

Line 349: Change 'we found none significant interaction' to 'we found no statistically significant interaction'.

WHODAS 2.0 results

Descriptive results

Line 358: It may be worth reminding the readers what MD, PD and FD refer to. At this point, I had to go back to the text and find out, interrupting the flow of the reading.

Lines 358 and 361: rho should have been defined. You give the symbol, but you have not defined it earlier.

Line 371-374: The figure caption is not very clear. When embedded in the text, it says 'Figure 2. Disability levels according to the WHODAS 2.0 domains and EQ-5D-5L dimensions, and to the sex. Results are shown from the lowest levels (on the left) to the highest levels (on the right).

Bar show the mean scores and standard deviations.'

However, when the figure captions are given in Lines 798 to 802, the text of the caption is different: ‘Figure 2. Disability levels according to the WHODAS domains and EQ-5D-5L dimensions, and to the sex and eye worm history. Results are shown from the lowest levels (on the left) to the highest levels (on the right)

Bar represent the means and standard deviations. a) global population study. b) black: male, grey: female. c) grey: none history of Ew, grey: more than 10 histories of Ew during their life’.

I cannot see panels (a), (b) and (c) when looking at the figure or downloading the TIF file. The figure seems incomplete. Also, change ‘and to the sex and eye worm (Ew) history’ to ‘by sex and eye worm history’. Where it says ‘Bar’ it should read ‘Bars and error bars represent, respectively, the means and standard deviations’. Where it says ‘none history of Ew’ it should read ‘no history of Ew’. Where it says ‘during their life’, it should say ‘during their lifetime’, for consistency with the Main Text. However, as previously pointed out, Figure 2 seems incomplete, with a single panel? Also, the upper error bars for the grey bars corresponding to ‘Mobility WHODAS’, ‘Household WHODAS’, ‘Daily EQ’, and ‘Pain EQ’ –the latter also for the black bar – do not seem to be displayed in their entirety, but capped at 70%. Please correct this figure and its caption.

Multivariable analyses

Line 415: ‘MD: mild disability’ is given as part of the caption of Figure 3, but when looking at Figure 3, I can only find ‘AMD’. Is this ‘Absent/missing data’? If so, the caption needs to be corrected. Also, when listing the Figure captions in Lines 796 to 802, the captions for Figures 3 and 4 are not given.

Line 417: Delete ‘Last’, so it reads ‘Only sex, age, …’

Line 418: Add ‘values’ after ‘adjusted Odds-Ratio (aOR)’ so it reads ‘adjusted Odds-Ratio (aOR) values were …’.

Line 421: Add ‘the’ before ‘model’ so it reads ‘Regarding the model ...’.

Line 423: Change ‘significantly retained in the final model’ to ‘statistically significant and, therefore, retained in the final model’.

EQ-5D-5L results

Descriptive results

Line 441: Delete ‘and’ after the semicolon.

Lines 439 and 442: Has rho been defined? See comment above.

Discussion

Line 513: Change ‘One should perhaps interpret this item with caution while waiting further …’ to ‘This item should be interpreted with caution while awaiting further ….’.

Line 515: Re-write ‘It is regrettable that Kato-Katz was used instead of more sophisticated techniques for diagnosing intestinal nematodes or protozoa, making it possible that a number of infections could not be identified.’ I suggest: ‘Kato-Katz was the only method used for diagnosing

intestinal infections, hampering our ability to detect other potential (protozoan and/or nematode) infections.’

Line 517: Add ‘status’ after ‘*T. trichiura* infection’, so it reads ‘*T. trichiura* infection status’, as for this parasite infection intensity was not measured.

Lines 524-525: Re-write ‘For example, it has been shown in a murine model infected with *T. muris* an increase in anxiety’ to read ‘For example, an increase in anxiety has been shown in a murine model of *T. muris* infection’.

Line 529: Change to ‘did not show statistically significant associations’.

Line 532: Replace the full-stop after Table 2 with a comma, so it reads ‘(Table 2, aIRR = 1.23)’.

Lines 542-543: Delete the determinate articles so it reads ‘were significantly associated with *L. loa* MFD but not with history of Ew episodes’.

Line 553: Add ‘detrimentally’ so it reads ‘was found to detrimentally impact mobility’.

Line 554: Change ‘poxy’ to ‘proxy’.

Line 566: Delete ‘*Loa*’ or change to ‘*L. loa* MFD’.

Line 567: Replace ‘impacted’ by ‘affected’.

Line 569: Should ‘associated with microfilariae’ be ‘associated with *L. loa* MFD’?

Line 575: Change ‘Conversely’ to ‘By contrast’.

Line 579: Delete ‘the’ so it reads ‘associated with loiasis status’.

Line 579: Replace ‘conversely to’ with ‘in contrast to’.

Line 593: Do you mean considerable disability rather than significant disability?

Line 616: Delete ‘Finally’ so it reads ‘While other potential explanatory factors’. There is a ‘Lastly’ in Line 620, so this cannot be ‘Finally’.

Acknowledgements

Line 652: Spell out PNLO or add ‘(PNLO)’ to line 19 (in affiliations).

Figures:

Lines 796-802: Titles and captions for Figures 3 and 4 are missing.

Supplementary Materials

The list seems incomplete or incorrect, as in the Main Text ‘S6 Figures’ and ‘S10 Figures’ are mentioned, but not listed? A suggestion to the authors (and in line with PLoS Negl Trop Dis guidelines) is to prepare a single Supplementary S1 File, and within it, name the items using letters. For example, Text A, Text B, Table A, Table B, Figure A, Figure B, etc. This will also avoid confusion

with the items of the WHODAS questionnaire, that also start with an ‘S’. To illustrate this, in Line 192, where it says ‘see appendix S1 Questionnaire’, you would say ‘see S1 File, Questionnaire A’ for the WHODAS questionnaire, and ‘S1 File, Questionnaire B’ for the EQ-5D-5L questionnaire (Lines 213-214). This will also help the reader to have all supplementary information items in one single Supporting Information S1 File.
