# Supplementary material for: Disability and quality of life assessment using WHODAS-12 items 2.0 and EQ-5D-5L in a rural area endemic for loiasis in the Republic of Congo: A population-based cross-sectional study (the MorLo project)
Source: PLoS Negl Trop Dis. 2025 Sep 15;19(9):e0013491. doi: 10.1371/journal.pntd.0013491 (PMC12449028; doi:10.1371/journal.pntd.0013491)
Supplement: Attachment — Submitted filename: Response to reviewers-WHODAS-Final.docx [file pntd.0013491.s015.docx]

**Editor comments**

Please ensure that the CRediT author contributions listed for every co-author are completed accurately and in full.

At this stage, the following Authors/Authors require contributions: Hemilembolo C Marlhand, Campillo T Jérémy, Dupasquier Valentin, Lebredonchel Elodie, Beneteau Samuel, Rancé G Ludovic, Madoulou Moulabou Glorifié, Niama Ange, Bileckot R Richard, Pion DS Sébastien, Boussinesq Michel, Cédric B. Chesnais, and Missamou François. Please ensure that the full contributions of each author are acknowledged in the "Add/Edit/Remove Authors" section of our submission form.
The list of CRediT author contributions may be found here: <https://journals.plos.org/plosntds/s/authorship#loc-author-contributions>

Response: done

2) We do not publish any copyright or trademark symbols that usually accompany proprietary names, eg ©, ®, or TM (e.g. next to drug or reagent names). Therefore please remove all instances of trademark/copyright symbols throughout the text, including:
- TM on page: 7.

Response: done

3) We notice that your supplementary Tables, and information are included in the manuscript file. Please remove them from the main file. Supporting files should be uploaded separately with the file type 'Supporting Information'. Please ensure that each Supporting Information file has a legend listed in the manuscript after the references list.

Response: done

4) We note that your Data Availability Statement is currently as follows: "Anonymized data will be hosted on the <https://dataverse.ird.fr/> server, and its terms of use will be those in force on the hosting site." Please confirm at this time whether or not your submission contains all raw data required to replicate the results of your study. Authors must share the “minimal data set” for their submission. PLOS defines the minimal data set to consist of the data required to replicate all study findings reported in the article, as well as related metadata and methods (<https://journals.plos.org/plosone/s/data-availability#loc-minimal-data-set-definition>).

Response: done

For example, authors should submit the following data:

1) The values behind the means, standard deviations and other measures reported;

2) The values used to build graphs;

3) The points extracted from images for analysis.

Authors do not need to submit their entire data set if only a portion of the data was used in the reported study.

If your submission does not contain these data, please either upload them as Supporting Information files or deposit them to a stable, public repository and provide us with the relevant URLs, DOIs, or accession numbers. For a list of recommended repositories, please see <https://journals.plos.org/plosone/s/recommended-repositories>. 
If there are ethical or legal restrictions on sharing a de-identified data set, please explain them in detail (e.g., data contain potentially sensitive information, data are owned by a third-party organization, etc.) and who has imposed them (e.g., an ethics committee). Please also provide contact information for a data access committee, ethics committee, or other institutional body to which data requests may be sent. If data are owned by a third party, please indicate how others may request data access.

5) Please amend your detailed Financial Disclosure statement. This is published with the article. It must therefore be completed in full sentences and contain the exact wording you wish to be published.
1) State what role the funders took in the study. If the funders had no role in your study, please state: "The funders had no role in study design, data collection and analysis, decision to publish, or preparation of the manuscript."

Response: done

**Reviewer 1:**

1. The study objectives—to assess quality of life and disability in a loiasis-endemic region—are clearly stated. However, the hypothesis regarding the different impact of adult worms compared to microfilarial density on QoL could have been described more explicitly in the manuscript.

Response: We thank the reviewer for this comment. We have added our hypothesis regarding the possible effects of both adult worms and microfilariae on QoL in the introduction. “Since microfilariae appear to be associated with increased mortality and severe organ complications, it is possible that microfilarial density (MFD) is linked to a globally impaired QoL, potentially reflecting an advanced state of fatigue resulting from specific complications (e.g., renal failure). Conversely, as no study to date has demonstrated an association between adult worms and severe complications, it is difficult to extrapolate their potential impact on QoL. However, a study conducted in Gabon found an association between loiasis status (defined as positive in the presence of a history of adult worms or microfilaremia) and general symptoms such as headaches, paresthesia, and arthralgia [4]. We hypothesize that adult worms, due to their migration, could contribute to symptoms related to chronic pain or mobility disorders, assuming a certain chronicity of these symptoms.” Furthermore, we have revised the penultimate paragraph of the discussion: “While studies have statistically demonstrated the negative repercussions of *L. loa* MFD on the functioning of certain human organs [5] and on population mortality rates [9,10], *L. loa* MFD does not seem to have a significant influence on disability in the population. Conversely, *L. loa* adult stages appear to significantly impair the population in their usual activities. Some authors reported that transient painful edema, arthralgia, myalgia, severe headache, transient paralysis of extremities, paresthesia, fatigue, and swellings of any body parts were more significantly associated with the loiasis status [4]. However, conversely to our study, *L. loa* adult worms’ history and microfilaremia were gathered to define loiasis status. Based on our results, it is likely that these various symptoms are primarily due to adult worms (as assessed by the Ew question) rather than the direct effect of microfilariae themselves. Apart from fatigue, which remains difficult to interpret, and transient edema, which most likely corresponds to Calabar swellings, we believe that the other reported symptoms could result from the migration of adult worms. In certain anatomical locations, where migration may be restricted due to structural constraints, adult worms could exert pressure on specific neural or vascular structures. For example, if a worm becomes trapped in a confined space, it could press on nerve sheaths or carpal tunnels, potentially leading to paresthesia in the extremities. Similarly, migration in the cervical region could contribute to occipital neuralgia (Arnold’s neuralgia), which may explain the observation of severe headaches. Additionally, worms migrating between intermuscular fasciae could be responsible for myalgia. Intra-articular migration, as previously suggested, could lead to synovitis. These mechanisms provide plausible explanations for the variety of symptoms observed in affected individuals. These clinical manifestations cause daily discomfort, leading to significant disability in affected individuals. These various results suggest that adult worms may have a clinical impact through peripheral impairments, whereas microfilaremia may have a clinical impact through severe organ involvement, potentially explaining why mortality has been associated only with *L. loa* MFD.”

2. The cross-sectional design is appropriate to the objectives, although it has inherent limitations for inferring causality. These limitations are correctly acknowledged by the authors.

Response: We thank the reviewer for this comment.

3. The target population is well-defined, with proper inclusion and exclusion criteria. Stratification of participants by microfilaremic status and symptom history aligns well with the research objectives. However, providing more details about how participants were recruited and addressing potential selection biases would improve the methodology section.

Response: We thank the reviewer for this comment. We added a flowchart on selection and recruitment procedure (now Fig. 1). We also added in the study design section: “Briefly, the inclusion criteria were: residency in the study area since 2019, age 18 or older, and prior screening for *L. loa* microfilaremia in 2019 as part of a participant selection survey for a previous clinical trial. Individuals with more than 500 *L. loa* microfilariae/mL in 2019 were matched by sex and age (within a five-year range) with two amicrofilaremic individuals from the same village.” We added a paragraph regarding limitations of this study.

4. The sample size of 991 participants seems sufficient. While power calculations and effect size estimations for the key outcomes (WHODAS and EQ-5D-5L scores) are mentioned, they could have been further elaborated to increase transparency.

Response: We thank the reviewer for this comment. We added these sentences in the power calculation section: “The sample size for the underlying cohort study had been calculated (991) to show an increase in the risk of malaria and pneumonia of x1.4 and x2.4 for microfilaremic vs amicrofilaremic individuals, with a power of 80%. In the present cross-sectional study, the sample size was not predetermined to detect differences in QoL due to a lack of prior data for calculation. However,…”.

5. The use of Poisson and Negative Binomial regression models is appropriate given the nature of the data. The manual backward selection process is valid, but the authors should explain better how they accounted for potential confounders and interactions.

Response: We thank the reviewer for this comment. Potential confounders were assessed in our multivariable models. We added interaction assessment in our method statistical section and results. “Finally, in all our models, we evaluated the potential effect of polyparasitism and with our main exposure variables using likelihood ratio tests for the following interaction terms: *A. lumbricoides* and *T. trichiura*, *A. lumbricoides* and *L. loa* MFD, *T. trichiura* and *L. loa* MFD, *A. lumbricoides* and Ew, *A. lumbricoides* and Calabar edema, *T. trichiura* and Ew, *T. trichiura* and Calabar edema, and last, a three-way interaction term between *A. lumbricoides*, *T. trichiura*, and Ew variables.” And “Finally, we found none significant interaction terms for all our models” in the modified section ‘Correlations, multicollinearity, and interaction terms’.

6. The handling of missing data (over 20% in some variables) requires a more explicit discussion to understand the impact on the findings.

Response: We do have a certain amount of missing data, particularly for stool examinations (219 cases, or 22.1%, for *Ascaris lumbricoides* and 217 cases, or 21.9%, for *Trichuris trichiura*), as well as for the Ew variable (173 cases, or 17.5%) and the Calabar variable (172 cases, or 17.4%). From a statistical perspective, it is possible to create a category for missing values to ensure the inclusion of the entire study population in our analyses. However, to address the reviewer’s concern more specifically, we have added the following statement in the results section: “Regarding the Ew/Calabar history group, individuals with missing values were significantly younger than respondents (median age: 45 vs. 53 years, p<0.001), were more likely to live alone rather than with a partner (46.5% vs. 34.4%, p=0.002), and were less likely to report agriculture as their primary occupation (67.8% vs. 80.9%, p<0.001). Conversely, they did not differ in terms of sex ratio (p=0.772), *L. loa* MFD (p=0.765), or years of education (p=0.623). Last, regarding the STH group, individuals with missing values were significantly younger than respondents (median age: 48 vs. 53 years, p=0.012), were more likely to live alone rather than with a partner (45.9 vs. 33.9%, p=0.001), and were less likely to report agriculture as their primary occupation (68.2% vs. 81.6%, p<0.001). Conversely, they did not differ in terms of sex ratio (p=0.660), *L. loa* MFD (p = 0.765), or years of education (p=0.958).” See the new paragraph for the limitations.

7. It would also be interesting to include details about the training of interviewers and the translation or adaptation process for the questionnaires

Response: We thank the reviewer for this comment. We added “These latter informations were collected by surveyors experienced in working with the National Onchocerciasis Control Program, and pictures were used, following the RAPLOA procedure [1]. » for Ew and Calabar assessment. In addition, we added: « The questionnaires were presented and discussed with local authorities and community health workers. This step was essential to ensure that certain terms were understandable to the population. Once completed, the two questionnaire supervisors were trained to administer the questions in a standardized manner. » in the disability and quality of life assessment section.

8. The conclusions are well-supported by the presented data. The novel finding that adult worms significantly impact QoL more than microfilarial density is important and contributes meaningfully to the field.

Response: We thank the reviewer for this comment.

9. The study’s limitations, including its reliance on self-reported symptoms and the cross-sectional design, are appropriately acknowledged. However, more discussion on how these limitations might influence the interpretation of results would be beneficial.

Response: We thank the reviewer for this comment. Here is the two new paragraphs gathered all our limitations, as suggested by the reviewers: “Several limitations to our study should be acknowledged. First, our initial screening procedure in 2019 was based on voluntary participation, which may have introduced a selection bias. However, if such a bias exists, it is likely to affect the overall representativeness of our study population rather than the relationship between our exposure variables and QoL. Second, as this is a cross-sectional study relying on retrospective self-reported data regarding past episodes of Ew and Calabar swelling, recall bias may be present. However, if recall bias exists, it is likely to be non-differential, meaning that it would primarily reduce the statistical power of our results rather than lead to misinterpretation. Importantly, since our participants are part of a longitudinal cohort, we will be able to assess the impact of loiasis on QoL more accurately through follow-up assessments in 2025 and 2028, which will include a reassessment of episode frequency within these intervals, thereby minimizing recall bias. Third, Although TBS is a gold standard technique and was prepared and read by highly experienced technicians in our study, we acknowledge that this method has a lower sensitivity compared to concentration techniques, which could result in the underestimation of microfilaremia prevalence for *L. loa*.

Additionally, our data contained a certain amount of missing data. To address this, we accounted for missing values by including them as a separate category in our models, allowing us to maintain statistical power. However, the observed results for this category may be partly explained by the fact that individuals with missing data were more frequently those who reported living alone—a variable that was itself often significantly associated with worse QoL indicators. Finally, while other potential explanatory factors for QoL, such as income level (as a proxy for socioeconomic status), could have been considered, we do not believe that residual confounding is a major concern, or at least, that any existing confounding would have a substantial impact. Nevertheless, the inclusion of such factors could have provided a more comprehensive understanding of QoL within our study population. Lastly, due to the very limited number of studies using WHODAS to assess QoL in the context of NTDs, drawing comparisons with existing literature remains challenging. However, as QoL is strongly influenced by local factors, our findings highlight the need for increased use of standardized tools like WHODAS in Central Africa, including for NTD-related research.”

10. The manuscript addresses an important gap in the understanding of QoL and disability in loiasis-endemic regions, highlighting the public health burden of the disease. The discussion connects the findings effectively to practical implications for patient care and policy-making

Response: We thank the reviewer for this comment.

11. The results are clearly presented, although some tables appear overly detailed. Consolidating information or combining variables into summary tables would enhance clarity for readers.

Response: We thank the reviewer for this comment. To make our results clearer, we decided to keep our tables; but we added in the manuscript a summary figure on our main result (IRR of the Ew variable for each of our WHODAS and EQ-5D-5L models), and we added as additional material all the final models in figure form.

12. Figures and tables are of good quality, but simplified labels and improved readability would further strengthen the presentation. For example, the term “MD” is used for both “missing data” and “mild disability,” which can confuse readers depending on the table or section.

Response: We thank the reviewer for this comment. We defined AMD as Absent/Missing Data; however, there was an error in one of our tables where it was mistakenly labeled as MD. We have corrected this to AMD. The term AMD appears only in the tables, accompanied by a footnote explanation, while MD, referring to mild disability, is used in the main text.

13. Statistical significance could also be visually highlighted in a more consistent manner across the figures

Response: We thank the reviewer for this comment. See response 11., we added several figures to easily visualize our results.

14. The manuscript is generally well-organized, but the following improvements are suggested to improve clarity and presentation:
    a. Improve the visual presentation of the results.
    b. Add subheadings in the results section for better organization.
    c. Optimize table formats to improve readability.
    d. Include a participant flow diagram to help readers understand how participants were selected and analysis process.

Response: We added several figures; but as explained, we are aware that we have several models. Given that we added several figures, we prefer keep our tables as currently in the manuscript. We added subheadings in the results section, and a flow diagram.

15. The use of standardized QoL tools such as WHODAS 2.0 and EQ-5D-5L is a significant strength, providing validated metrics for cross-study comparisons.

Response: We thank the reviewer for this comment.

16. The large sample size improves the generalizability of the findings within the loiasis-endemic region.

Response: We thank the reviewer for this comment.

17. However, recall bias may have influenced the self-reported outcomes, such as Calabar swellings and worm passage history. Additionally, the absence of a discussion on socioeconomic factors, which could confound QoL and disability results, could be a relevant limitation.

Response: We thank the reviewer for this comment. We added one paragraph for all our limitations. See the new paragraph for the limitations.

18. This manuscript represents a valuable addition to the field, offering insights into the QoL impact of loiasis and bridging a knowledge gap in NTD and disability research.

Response: We thank the reviewer for this comment.

19. It is suggested to expand the discussion of potential mechanisms, provide additional context on prior QoL studies in NTDs, further elaborate on the implications for control programs, and strengthen the discussion on the unexpected *T. trichiura* findings.

Response: We thank the reviewer for this comment.

- We have expanded the discussion on potential mechanisms to explain the differential results observed between adult worms and microfilariae (see previous response).
- Regarding the unexpected *T. trichiura* findings, we added “Finally, we cannot exclude a possible problem of interpretation on the part of the participants, as infection with T*. trichiura* has sometimes been described as being associated with 'nervousness' [32,33].” However, the existing information on anxiety is scarce and comes exclusively from experimental animal studies. As stated in the discussion, this result warrants further investigation, but due to the lack of additional references, we cannot further extrapolate potential mechanisms. However, we believe this finding should be highlighted as a potential signal of such an association.
- Currently, there are no control programs for loiasis. As such, elaborating on the direct implications of our findings in this manuscript would be premature. At this stage, our results primarily serve to guide future research—particularly in understanding the respective roles of adult worms and microfilariae—and to provide scientific evidence supporting the recognition of loiasis as a Neglected Tropical Disease.
- Finally, very few similar studies have been conducted on NTDs using the same questionnaire, and our study is the first on loiasis. As mentioned in the introduction, previous studies include one on Buruli ulcer in Ghana, one on podoconiosis in Ethiopia, one on onchocerciasis in Nigeria, and two on lymphatic filariasis (but not in Africa). We believe that QoL studies are highly dependent on local context (geographical, economic status, living conditions, rural vs. urban settings, etc.). Therefore, drawing direct comparisons between our findings in a rural area of the Republic of Congo and those from studies conducted in entirely different contexts, with different infections, would be challenging and potentially misleading. Please refer to the newly added paragraph in the limitations section for further clarification.

**Reviewer 2:**

1. This is an important study of one of a most common parasitic disease in Central Africa. Study on quality of life as well as disability in case of loiasis had not yet been carried out. Although the thematic is of importance; major issues in study design, as well as in the analysis of the data, require major revisions before its consideration for publication.

Response: We thank the reviewer for this comment.

**Methods**

2. Although information on the study villages is presented elsewhere, it is important to specify the known endemicity level of loiasis in order to have an idea of the exposure of the populations.

Response: We thank the reviewer for this comment. We added “(overall prevalence of 20% for microfilaremia [21])” in the study design section.

3. Moreover, although they are rural populations, their main income-generating activities are not specified, this can have an influence on the responses to the various questionnaires used.

Response: We thank the reviewer for this comment. We added two paragraphs on our all limitations. “Several limitations to our study should be acknowledged. First, our initial screening procedure in 2019 was based on voluntary participation, which may have introduced a selection bias. However, if such a bias exists, it is likely to affect the overall representativeness of our study population rather than the relationship between our exposure variables and QoL. Second, as this is a cross-sectional study relying on retrospective self-reported data regarding past episodes of Ew and Calabar swelling, recall bias may be present. However, if recall bias exists, it is likely to be non-differential, meaning that it would primarily reduce the statistical power of our results rather than lead to misinterpretation. Importantly, since our participants are part of a longitudinal cohort, we will be able to assess the impact of loiasis on QoL more accurately through follow-up assessments in 2025 and 2028, which will include a reassessment of episode frequency within these intervals, thereby minimizing recall bias. Third, Although TBS is a gold standard technique and was prepared and read by highly experienced technicians in our study, we acknowledge that this method has a lower sensitivity compared to concentration techniques, which could result in the underestimation of microfilaremia prevalence for *L. loa*.

Additionally, our data contained a certain amount of missing data. To address this, we accounted for missing values by including them as a separate category in our models, allowing us to maintain statistical power. However, the observed results for this category may be partly explained by the fact that individuals with missing data were more frequently those who reported living alone—a variable that was itself often significantly associated with worse QoL indicators. Finally, while other potential explanatory factors for QoL, such as income level (as a proxy for socioeconomic status), could have been considered, we do not believe that residual confounding is a major concern, or at least, that any existing confounding would have a substantial impact. Nevertheless, the inclusion of such factors could have provided a more comprehensive understanding of QoL within our study population. Lastly, due to the very limited number of studies using WHODAS to assess QoL in the context of NTDs, drawing comparisons with existing literature remains challenging. However, as QoL is strongly influenced by local factors, our findings highlight the need for increased use of standardized tools like WHODAS in Central Africa, including for NTD-related research.”

4. L92: how was estimate of Fatigue measured.

Response: We thank the reviewer for this comment. We used this term because the study published in The Lancet Infectious Diseases (Veletzsky et al.) reported an association between loiasis status and fatigue. However, the authors did not specify how this symptom was assessed in their publication.

5. L125-126: Deworming campaigns target children not included in the study population.

Response: We thank the reviewer for this comment. We dropped these words.

6. The authors should better justify the relevance of these campaigns in the design of the study. In addition, information on the molecules used for chemoprevention shall be provided.

Response: We thank the reviewer for this comment. We dropped these words. This information ultimately seems irrelevant to our analyses. The existence of a campaign targeting children does not reflect the reality of these infections in adults, nor does it affect disease progression in this population. Therefore, envision a relationship or useful information from deworming campaigns in children population has no impact on the quality of life in our adult population.

7. The tests used for loiasis detection are also of concern
   a. L132: why did the direct examination of 10μL of blood not examined?

Response: We have decided to use 50 µL TBS to assess *Loa loa* microfilaremia in the study participants. We opted for a single measurement, consistent across all individuals, rather than multiple measurements (such as by concentration or direct examination of 10µL, for example) to ensure reproducibility and comparability

b. What is the sensitivity of the 50μL Giemsa stained Thick smear?

Response: The most sensitive technique would be the concentration method. However, no study has compared the commonly used technique that we applied with the concentration method, making it impossible to determine its exact sensitivity. Nevertheless, it is possible that, in very rare cases, some individuals classified as amicrofilaremic may have had a few undetected microfilariae. We have added the following limitation to the new paragraphs on limitations.

c. What is the sensivity and specicity of the *Loa loa* RDT. Cross-reactivities have always been described? How were the participants without microfilaremia with a positive RDT?

Response: We thank the reviewer for this comment. There are currently no available data on the specificity of this test. Regarding sensitivity, it is estimated at 84% and was determined within the same study population (Campillo et al., Evaluation of a novel biplex rapid diagnostic test for antibody responses to *Loa loa* and *Onchocerca volvulus* infections, PLOS NTD, 2024). This is a new test, and its interpretation remains challenging in an endemic context for loiasis. Its utility is likely greater for large-scale applications, such as mapping campaigns or excluding *Loa loa* infection in non-endemic areas, rather than for characterizing individual infections and assessing their severity. Given the current length of the discussion and the lack of association for this variable, we believe it is unnecessary to include it in this manuscript.

8. Why are HbAS or HbSS patients individualized for data analysis. Why are other subpopulations such as malnourished people, those in particular occupations not?

Response: We thank the reviewer for this comment. We had no patients with HbSS. Moreover, no studies have assessed QoL in heterozygous individuals (HbAS vs HbAA), and our results show no association with our QoL indicators (see Supplementary Material). Similarly, BMI was included as stated in the methods, and we found no significant association (results available in the full models in the Supplementary Material).

9. L115-162: the authors have used only Kato-katz for the detection of intestinal parasites. This technique does not make it possible to properly detect all the parasitic stages of the protozoa, which themselves are likely to be responsible for nonspecific but suggestive symptoms of loiasis and responsible for disability

Response: We thank the reviewer for this comment. *Ascaris lumbricoides* and *Trichuris trichiura* are nematodes. We did not employ sufficient techniques to identify different stages for protozoa (amoebae for example in the case of hepatic amoebiasis with serology or *Gardia duodenalis* for example; although a simple examination by Kato-Katz can highlight amoeba eggs). We added in the discussion: « It is regrettable that Kato-Katz was used instead of more sophisticated techniques for diagnosing intestinal nematodes or protozoa, making it possible that a number of infections could not be identified. However, an intriguing result… ».

10. L168: please explain why "Lifetime"

Response: We thank the reviewer for this comment. Loiasis is a cumulative and chronic infection. Therefore, we chose to prioritize assessing lifetime exposure to *Loa loa* rather than focusing solely on the past year.

11. L164-170: Explain how the symptoms were assessed? Calabar swellings are shown to do not be inflammatory, neither painful (unless extended) and to last several days.

Response: We thank the reviewer for this comment. We added: “These latter informations were collected by surveyors experienced in working with the National Onchocerciasis Control Program, and pictures were used, following the RAPLOA procedure”.

12. The study population with microfilariae most likely has a chronic infection. the majority of these patients describe passages of a worm in the eye. A patient may have several episodes per day within a week. The authors should further describe the modalities of clinical evaluation and number of episodes throughout the year assessment and stratification. Who administered the questionnaires, how was it administered so that ALL the participants, could understand the illustrations and questions.

Response: We thank the reviewer for this comment. But before providing a more detailed response, the Ew and Calabar episodes were collected through a questionnaire about past episodes, rather than being clinically assessed based on current events.

- In fact, we already have a paper accepted for publication in Plos NTD on the incidence of these symptoms with prospective work over one year on episodes of Ew and Calabar; taking into account everything you suggest. Here, as we presented in the document, and as we assume that this is precisely a chronic infection, we have used the Ew and Calabar episodes during lifetime to assess QoL. Given the complexity of the analyses and the number of models, we believe it is preferable to keep this structure simpler, by simply differentiating between the microfilarial density variable and the lifetime histories of Ew and Calabar, independently. In addition, we checked our correlations, our interactions, and a possible multicollinearity problem; the results of which indicate that, statistically, we can proceed in this way. Finally, by stratifying, we would have assumed that there would have been interaction effects, which was not the case.
- We have added « The questionnaires were presented and discussed with local authorities and community health workers. This step was essential to ensure that certain terms were understandable to the population. Once completed, the two questionnaire supervisors were trained to administer the questions in a standardized manner. »

13. Were there any awareness sessions or information during which loiasis symptoms were described, prior to the investigation?

Response: We thank the reviewer for this comment. Our team has been working in these communities for 5 years, with a series of screening sessions. With the support of the local authorities and community health workers, several sessions have been held to explain the disease and in particular the characteristics of Ew and Calabar oedema. However, despite these awareness-raising sessions and the support provided by pictures during this study, Calabar oedema may be subject to possible differential diagnoses, implying that certain declarations, particularly for this symptom, may be false.

14. There is also an ethical issue: there is no indication of participants treatment during the one year follow-up. Does it mean that infected participants as well as the symptomatic ones remained untreated during one year? What was the ethics committee recommendation regarding these aspects?

Response: We thank the reviewer for this comment. In endemic areas, there is currently no recommendation to treat individuals infected with loiasis. Only national programmes have the authority to treat the population, and then only as part of mass treatment campaigns for onchocerciasis and lymphatic filariasis. Loiasis is not recognized as a NTD and does not benefit from a control programme. In these areas, ivermectin use is also restricted (only programmes have access to it). Consequently, we cannot offer individual treatment for loiasis. However, and this is why our team is working on this issue, we are conducting clinical trials to find solutions that are acceptable to the population. We hope to be able to break the deadlock quickly, so that the people of Central Africa can benefit from treatment. The ethics committee is aware of these issues.

**Results**

15. L282-287: a study flow chart would be more appropriate as only 771 out of the 990 or 987 participants were tested for intestinal parasite carriage

Response: We thank the reviewer for this comment. We added a flowchart (now Fig 1).

16. What is the significance of analyzing the STH carriers when the main symptoms used to assess disability and quality of life are specific for loiasis.

Response: We thank the reviewer for this comment. The WHODAS and EQ-5D-5L are not specific loiasis questionnaires. As we stated in the introduction and the method, they are standardized questionnaires validated by several studies and by the WHO to assess QoL, whether for non-infectious or infectious diseases. Thus, whether for STH, loiasis, or our other assessed factors, these questionnaires enable QoL to be assessed for each of these factors, without presupposing a certain added value in terms of expected symptoms.

17. On the other hand, associated symptoms such as generalized or localized pruritus, photophobia, are most likely to be associated with disability if chronic. These are the most frequent reasons for patient consultation. Pruritus is also a symptom described during certain STH and pathogenic c intestinal protozoa carriage. The fact that half of the participants tested carry at least one STH and that the protozoa have not been tested, should be better discussed.

Response: We thank the reviewer for this comment. None of the questions in the questionnaires used (see Supplementary material and answer 22) relate to pruritus or photophobia. Furthermore, as mentioned above, no association between our different parasites on our QoL indicators was significant. We added in the discussion: « It is regrettable that Kato-Katz was used instead of more sophisticated techniques for diagnosing intestinal nematodes or protozoa, making it possible that a number of infections could not be identified. However, an intriguing result… ».

18. Patients should be stratified according to the type of parasitism, and data from the questionnaire presented accordingly; i.e. absence of parasite, single parasite carriage, multiple parasite carriage (several microFilariae, *Loa loa* +STH).

Response: We thank the reviewer for this comment. We added interaction assessment in our method statistical section and results. “Finally, in all our models, we evaluated the potential effect of polyparasitism and with our main exposure variables using likelihood ratio tests for the following interaction terms: *A. lumbricoides* and *T. trichiura*, *A. lumbricoides* and *L. loa* MFD, *T. trichiura* and *L. loa* MFD, *A. lumbricoides* and Ew, *A. lumbricoides* and Calabar edema, *T. trichiura* and Ew, *T. trichiura* and Calabar edema, and last, a three-way interaction term between *A. lumbricoides*, *T. trichiura*, and Ew variables.” And “Finally, we found none significant interaction terms for all our models” in the modified section ‘Correlations, multicollinearity, and interaction terms’. In other words, to assess the effects of multiparasitism, we prioritized this approach as it allowed us to evaluate the potential impact of co-infections while still analyzing the results for each parasite individually (rather than pooled). This also ensured sufficient statistical power for our analyses, which stratified analyses would not have allowed. However, given the interaction tests, these stratifications would not ave been justified anyway.

**Discussion**

19. The discussion should be amended after considering all the comments above.

Response: Done.

**MINOR COMMENTS**

20. L278: test for age distribution and choose mean or median, there is no need to present both

Response: We thank the reviewer for this comment. We modified it.

21. L287-290: the global prevalence of STH carriers should be presented. 50.9% of the tested participants had an STH infection, this is higher than Loiasis prevalence

Response: We do not understand the reviewer's comment. Indeed, as indicated, we have already reported, for descriptive purposes only, that 50.7% (and not 50.9%) were positive for at least one STH. For the rest of the document, and as explained above, we have no justification for wanting to group the STH category into a single category.

22. L445: which symptom was painful?

Response: We thank the reviewer for this comment. We don't have an answer. To go back to the methodology. On the one hand, we asked for the number of episodes of Ew and Calabar oedema; and we collected information to also assess our other exposure factors whose effect on QoL we wished to evaluate (BMI, smoking,..., STH, *L. loa* microfilarial density). On the other hand, we applied the standardised WHODAS 2.0 and EQ-5D-5L questionnaires as presented in the supplementary material. These are specific questions and no other questions are used. We applied the conditions of application of these questionnaires. For example, for this question on pain, the question was simply "First, I'm going to read you some questions. Each question offers a choice of five answers. Please tell me which answer best describes your health TODAY. Question PAIN : I'd like to ask you about PAIN(S) or discomfort. Would you say that: you have no pain or discomfort? you have slight pain or discomfort? you have moderate pain or discomfort? you have severe pain or discomfort? Our indicators therefore do not correspond to specific symptoms and are not intended to describe or characterize Ew or Calabar oedema in our population, but simply to provide an overall assessment tool for QoL and to see whether or not one of the exposure factors is significantly associated with worse or no QoL scores.
