# Supplementary material for: Disability and quality of life assessment using WHODAS-12 items 2.0 and EQ-5D-5L in a rural area endemic for loiasis in the Republic of Congo: A population-based cross-sectional study (the MorLo project)
Source: PLoS Negl Trop Dis. 2025 Sep 15;19(9):e0013491. doi: 10.1371/journal.pntd.0013491 (PMC12449028; doi:10.1371/journal.pntd.0013491)
Supplement: Attachment — Submitted filename: Response_to_reviewers_WHODAS_R2.docx [file pntd.0013491.s016.docx]

**Responses to reviewers PNTD-D-24-01363R1**

**Disability and quality of life assessment using WHODAS-12 items 2.0 and EQ-5D-5L in a rural area endemic for loiasis in the Republic of Congo: a population-based cross-sectional study (the MorLo project)**

**Journal Requirements:**

1) We have noticed that you have uploaded Supporting Information files, but you have not included a complete list of legends. Please add a full list of legends for your Supporting Information files after the references list. Please ensure that the labels of the supplementary files match their citations and legends. For example S4 table is labeled as Appendix S4 and S10 Figure is labeled as Appendix S10.

**Response**: Done, we added Appendix after each S[X] and harmonized in the main text.

2) Regarding Figure 1, thank you for stating "Adapted from 7." Please include the source details in the figure legend.

**Response**: We added source details in the figure legend.

3) We note that you have indicated that there are restrictions to data sharing for this study. PLOS only allows data to be available upon request if there are legal or ethical restrictions on sharing data publicly. For more information on unacceptable data access restrictions, please see <https://journals.plos.org/plosntds/s/data-availability#loc-unacceptable-data-access-restrictions>.
Before we proceed with your manuscript, please address the following prompts:
a) If there are ethical or legal restrictions on sharing a de-identified data set, please explain them in detail (e.g., data contain potentially identifying or sensitive patient information, data are owned by a third-party organization, etc.) and who has imposed them (e.g., a Research Ethics Committee or Institutional Review Board, etc.). Please also provide contact information for a data access committee, ethics committee, or other institutional body to which data requests may be sent.
b) If there are no restrictions, please upload the minimal anonymized data set necessary to replicate your study findings to a stable, public repository and provide us with the relevant URLs, DOIs, or accession numbers. For a list of recommended repositories, please see <https://journals.plos.org/plosone/s/recommended-repositories>. You also have the option of uploading the data as Supporting Information files, but we would recommend depositing data directly to a data repository if possible.
We will update your Data Availability statement on your behalf to reflect the information you provide.

**Response**: Our department responsible for data sharing regulations has indicated that our data are pseudonymized (age and sex). Therefore, in addition to the online DOI, applicants are required to complete a data access agreement form. The DOI link will thus lead to both the dataset and the form to be completed for access.

**Reviewer 1:**

I appreciate the authors' effort in addressing the recommendations from the initial review. The revisions have significantly improved the clarity and methodological rigor of the manuscript, particularly in explicitly stating the hypothesis, providing more details on participant selection, and handling missing data. The inclusion of new figures and a flowchart has enhanced the readability of the results, while the expanded discussion section has provided a more comprehensive analysis of the findings, especially regarding the differential impact of adult worms and microfilarial density on quality of life. Given these improvements, I consider the manuscript suitable for publication.

**Response**: We sincerely thank the reviewer.

**Reviewer 2: N/A**

**Reviewer 3:**

This paper, submitted in 2024, has already been refereed by two competent reviewers and the authors have prepared a point-by-point reply to these referees. I am not an expert in Quality of Life assessments, but the paper is important, and I read it with interest. The authors prepared a revised version that addressed the comments by the previous reviewers, so I do not have further comments on the Objectives, Methods, Results and Conclusions. However, some text can be improved for clarity and conciseness, as well as for grammar and punctuation. In the below, I list my comments by section of the paper, and by line. Figures and figure captions need some attention, as well as the supplementary materials (which I suggest be consolidated into a single S1 File, and referred to by (capital) letters rather than by numbers, following PLoS Negl Trop Dis guidelines when preparing a single Supporting Information file. I give some explanation as to how this can be done. The authors would need to change the way they refer to Supporting Information items in the Main Text, which in any case also requires some attention, as it is currently inconsistent.

**Response**: We thank the reviewer for all the numerous corrections that have improved the manuscript. We have responded to each point in the following sections of the document.

**Abstract**

1. Line 39: Where it says ‘Calabar swellings episodes’ it should say ‘Calabar swelling episodes’ (as adjectives should be singular’.

**Response**: We thank the reviewer for this correction.

**Author Summary**

2. Line 64: Where it says ‘Conversely’, change to ‘By contrast’.

**Response**: Done.

**Introduction**

3. Line 78: Where it says ‘*Chrysops* sp.’ It should say ‘*Chrysops* spp.’ (as the authors are referring to more than one species).

**Response**: We thank the reviewer for this correction.

4. Line 78: Where it says ‘from southeast Benin …’, the authors should provide a reference after

**Response**: We thank the reviewer for this careful correction. We have added the suggested reference.

5. ‘Angola’, as the paper by Zouré et al. does not include Benin, and RAPLOA surveys may not have been conducted there. Please cite Boussinesq & Gardon (1997) Prevalences of *Loa loa* microfilaraemia throughout the area endemic for the infection, published in Ann Trop Med Parasitol.

**Response**: We thank the reviewer for this careful correction. We have added the suggested reference.

6. Line 81: Delete the determinate article ‘the’ before subconjunctival so it reads: ‘such as pruritus, subconjunctival migration of the adult worms (“eye worm”), …’.

**Response**: Done.

7. Lines 82-83: Change the semicolon to a comma and ‘would be’ to ‘is’, and add ‘medical’ before ‘consultation’, so the sentence reads: ‘, ‘and is a frequent reason for medical consultation’. Would be is tentative, but as you are citing papers, this is not your conjecture.

**Response**: We thank the reviewer for this careful correction.

8. Lines 83-84: Change ‘could have a significant public health impact’ to ‘has been reported to have a considerable public health impact’. Again, ‘could have’ is speculative, but then you are citing a paper quantifying the disease burden in Gabon. Also, reserve the use of ‘significant’ for ‘statistically significant’ throughout the paper to avoid any confusion.

**Response**: Done.

9. Lines 84-85: add ‘of disease’ to ‘burden’ (because in other contexts burden is also used for worm burden, and you are specifically referring to the disease burden here). I suggest to re-write the sentence as follows: ‘Based on these symptoms alone, loiasis has been reported to have a considerable public health impact, with its estimated burden of disease in Gabon being close to that of schistosomiasis.’

**Response**: Done.

10. Line 88: Add ‘progeny’ to larval’ (because there are other larval stages in the life-cycle (for instance within an infected/infective vector), so it reads: ‘larval progeny stages of the parasite’.

**Response**: Done.

11. Line 91: Change ‘impact’ to ‘detrimentally affect’ so it reads ‘some manifestations might detrimentally affect everyday activity’. In this way you also avoid the overuse of the word ‘impact’.

**Response**: Sincerely thank for all these precious corrections.

12. Line 94: Change ‘microfilarial density (MFD) is linked to a globally impaired QoL’ to ‘microfilarial density (MFD) is linked to an overall impaired QoL’. At this stage you have not yet introduced the global score terminology.

**Response**: Done.

13. Lines 95-97: The word ‘Conversely’ means ‘the opposite’, which I don’t think conveys the message well. I suggest to change to ‘Furthermore’. Also change ‘potential impact’ to ‘potential effect’ so it reads: ‘Furthermore, as no study to date has demonstrated an association between adult worms and severe complications, it is difficult to extrapolate their potential effect on QoL.’

**Response**: Done.

14. Lines 100-101: I don’t think that starting the sentence with ‘We hypothesize that …’ is necessary, because you are using ‘could contribute’ as a conjecture. Also, ‘assuming a certain chronicity of these symptoms’ could be omitted (as you are already mentioning ‘chronic pain or mobility’. I suggest to simplify this sentence to read: ‘Adult worms, due to their migration, could contribute to symptoms related to chronic pain or mobility disorders’ for clarity and conciseness.

**Response**: Done.

**Study design**

15. Line 129: Add ‘the’ to ‘baseline’, because you are referring to a particular cohort, so it reads: ‘It was conducted at the baseline of the cohort from May 16 to June 11, 2022’.

**Response**: Done.

16. Line 131: Add ‘of the Republic of Congo’ to ‘Lékoumou division’, so it reads: ‘Lékoumou division, of the Republic of Congo’.

**Response**: Done.

17. Line 132: Delete the full stop after 20 so it reads: ‘prevalence of 20%’. Add ‘and’ and change ‘hypo- meso endemic’ to ‘hypo- to mesoendemic’ so it reads: ‘and hypo-to mesoendemic for genito-urinary schistosomiasis’.

**Response**: Done.

18. Line 135: Delete ‘the’ before ‘Congo’. Nowhere else in the paper this is used, so it reads: ‘Republic of Congo’.

**Response**: Done.

19. Line 137: The text mentions a previous clinical trial, but no reference is given. Please give a reference here to a published paper or to the registration of this clinical trial.

**Response**: Done.

**Laboratory procedures**

20. Line 158: Add ‘The’ before ‘incubation medium’, so it reads: ‘The incubation medium was examined’.

**Response**: Done.

21. Line 163: Change ‘Last’ to ‘Also,’ so it reads: ‘Also, we performed a urinalysis’. You have a ‘Finally’ later (in Line 168), so this cannot be ‘Last’.

**Response**: Done.

22. Line 169: You have not yet defined STH. Please change to: ‘stool samples for soil-transmitted helminthiasis (STH) and intestinal schistosomiasis screening’.

**Response**: Done.

**Disability and quality of life assessment**

23. Line 204: Change ‘First’ to ‘Firstly’, as the former is used in informal writing and speech, while ‘firstly’ is more formal.

**Response**: Done.

24. Line 214: Give a reference for the "eq5d" R package, as follows: Morton F, Nijjar JS. Calculating EQ- 5D indices and summarising profiles with eq5d. 2025. Available from: [https://cran.r-](https://cran.r-project.org/web/packages/eq5d/vignettes/eq5d.html) [project.org/web/packages/eq5d/vignettes/eq5d.html](https://cran.r-project.org/web/packages/eq5d/vignettes/eq5d.html).

**Response**: Done.

**Power calculation**

25. Line 231: The reasoning behind ‘The sample size for the underlying cohort study had been calculated (991) to show an increase in the risk of malaria and pneumonia of x1.4 and x2.4 for microfilaremic vs amicrofilaremic individuals’ is not entirely clear. Please clarify why x1.4 and x2.4 were used.

**Response**: We thank the reviewer for this helpful comment. Our prospective cohort was designed to include one microfilaremic individual for every two amicrofilaremic individuals, identified through a large-scale screening campaign within a 50 km radius of our study site. This screening allowed us to identify approximately 330 mf+ and 660 mf− individuals, and all microfilaraemic individuals who consented were included. At the time of writing the MorLo project—based on a 3-year general population cohort—we considered this as our eligible study population. The primary objective was to assess whether microfilaremic individuals had a higher incidence of infectious diseases compared to amicrofilaremic individuals during follow-up. Assuming a malaria incidence of 200/1,000/year (https://map.ox.ac.uk/trends/country/COG), we expected at least 400 cases over the project duration. With this sample size, the statistical power to detect a hazard ratio (HR) of 2.0 for malaria was 100%, and 80% for an HR of 1.4. For pneumonia (expected incidence of 10/1,000/year), our study had 80% power to detect an HR of 2.4. In other words, these risk estimates correspond to the minimum detectable effects with 80% power, the standard threshold used in epidemiological research.

Here is the revised beginning of the sample size section: “The study cohort was established by including all identified microfilaremic individuals (approximately 330) within a 50 km radius around Sibiti, and selecting two amicrofilaremic individuals for each microfilaremic participant, resulting in a total sample of about 990 individuals. This sample size allowed sufficient statistical power to address the primary objective of the cohort: to detect with a 80% power an increased incidence of infectious diseases among microfilaremic compared to amicrofilaremic individuals over a 3-year follow-up period.”

26. Line 236: A global WHODAS score >25 is referred to as indicating ‘moderate disability’. But later on (Line 279), it is said that a score >50 is indicative of ‘severe global disability’. Should this moderate disability be >25 to 50? I am not a WHODAS expert, but other readers may not be either, so worth clarifying?

**Response**: We thank the reviewer for this clarification, which highlights that our initial wording was imprecise. A WHODAS score above 25 reflects moderate disability, while a score above 50 indicates severe disability. We acknowledge that we misused the terminology and have now corrected the text to state “at least moderate disability” when referring to scores >25.

27. Line 256: Add reference to the "psych" package in R, as follows: Revelle W. psych: procedures for psychological, psychometric, and personality research. 2025. Available from: [https://cran.r-](https://cran.r-project.org/web/packages/psych/index.html) [project.org/web/packages/psych/index.html](https://cran.r-project.org/web/packages/psych/index.html).

**Response**: Done.

**Disability assessment**

28. Line 277: Change ‘variable’ to ‘variable(s)’.

**Response**: Done.

29. Line 278: Change ‘significant’ to ‘statistically significant’. Line 278: Change ‘Last’ to ‘Finally’.

**Response**: Done.

30. Lines 279-280: Where it says: ‘…score >25 (moderate global disability – or significant disability) and >50 (severe global disability)’, is there an intermediate value missing for significant disability? Is a score >25 to 50 indicative of moderate disability or significant disability? See also question in Line 236 above. I was confused about this and other readers may be too. Worth clarifying if possible?

**Response**: See response 26; we modified all the terms in the manuscript.

31. Line 283: Change ‘variable’ to ‘variable(s)’.

**Response**: Done.

32. Line 287: ‘Given that we followed the instructions to analyze these questionnaires’, which instructions are these precisely? Not very clear.

**Response**: The analysis of these questionnaires was performed according to our pre-specified analysis plan and following the recommended guidelines: Ustun TB, Kostanjsek N, Chatterji S, Rehm J. Measuring Health and Disability - Manual for WHO Disability Assessment Schedule WHODAS 2.0 (World Health Organization, 2010), and The EuroQol Group (1990). EuroQol - a new facility for the measurement of health-related quality of life. Health Policy, 16(3):199-208; We also referred directly to the official guidelines available on each questionnaire’s website. However, since this information is already cited after each questionnaire’s description, we felt this part of the sentence was redundant and have removed it.

33. Line 288: ‘… significant number of scores evaluated’. Do you mean considerable? Statistically significant Please clarify.

**Response**: Done, we replaced with substantial.

34. Line 299: Delete ‘last’ so it reads ‘and a three-way interaction term …’.

**Response**: Done.

**Description of the study population**

35. Line 309: Change ‘year of schooling’ to ‘years of schooling’. Line 315: Change ‘sp.’ to ‘spp.’

**Response**: Done.

36. Line 317: Change ‘positive at’ to ‘positive for’.

**Response**: Done.

37. Line 324: Delete ‘Conversely’ so it reads ‘They did not differ in terms of sex ratio …’. Line 325: Delete ‘Last’ so it reads: ‘Regarding the STH group …’.

**Response**: Done

38. Line 329: Delete ‘Conversely’ so it reads ‘They did not differ in terms of sex ratio …’.

**Response**: Done.

**Internal consistency of the questionnaires**

39. Lines 333-335: Change ‘with a total omega coefficient of 0.9, a correlation of scores with a factor of 0.97 and a multiple R square of scores with a factor of 0.94’ to ‘with a total omega coefficient equal to 0.9, a correlation of scores with a factor equal to 0.97, and a multiple R square of scores with a factor equal to 0.94’. I think this makes the meaning clearer.

**Response**: Done.

**Correlations, multicollinearity, and interaction terms**

40. Line 342: Since you are starting a new paragraph, write *Trichuris* in full. Line 348: Clarify if you mean statistically significant correlations.

**Response**: We replace *T.* with *Trichuris*, and added statistically.

41. Line 349: Change ‘we found none significant interaction’ to ‘we found no statistically significant interaction’.

**Response**: Done.

**WHODAS 2.0 results Descriptive results**

42. Line 358: It may be worth reminding the readers what MD, PD and FD refer to. At this point, I had to go back to the text and find out, interrupting the flow of the reading.

**Response**: Done.

43. Lines 358 and 361: rho should have been defined. You give the symbol, but you have not defined it earlier.

**Response**: We added it in the method section.

44. Line 371-374: The figure caption is not very clear. When embedded in the text, it says ‘Figure 2. Disability levels according to the WHODAS 2.0 domains and EQ-5D-5L dimensions, and to the sex. Results are shown from the lowest levels (on the left) to the highest levels (on the right). Bar show the mean scores and standard deviations.’

**Response**: We apologize for the misunderstanding. The current figure only considers sex; the previous label at the end of the document was incorrect. We have now updated the title to: Disability levels by sex according to the WHODAS 2.0 domains and EQ-5D-5L dimensions. Note: Bars and error bars represent, respectively, the means and standard deviations. We have also revised the figure to include the four bars that were previously missing.

45. However, when the figure captions are given in Lines 798 to 802, the text of the caption is different: ‘Figure 2. Disability levels according to the WHODAS domains and EQ-5D-5L dimensions, and to the sex and eye worm history. Results are shown from the lowest levels (on the left) to the highest levels (on the right). Bar represent the means and standard deviations. a) global population study. b) black: male, grey: female. c) grey: none history of Ew, grey: more than 10 histories of Ew during their life’. I cannot see panels (a), (b) and (c) when looking at the figure or downloading the TIF file. The figure seems incomplete. Also, change ‘and to the sex and eye worm (Ew) history’ to ‘by sex and eye worm history’. Where it says ‘Bar’ it should read ‘Bars and error bars represent, respectively, the means and standard deviations’. Where it says ‘none history of Ew’ it should read ‘no history of Ew’. Where it says ‘during their life’, it should say ‘during their lifetime’, for consistency with the Main Text. However, as previously pointed out, Figure 2 seems incomplete, with a single panel? Also, the upper error bars for the grey bars corresponding to ‘Mobility WHODAS’, ‘Household WHODAS’, ‘Daily EQ’, and ‘Pain EQ’ –the latter also for the black bar – do not seem to be displayed in their entirety, but capped at 70%. Please correct this figure and its caption.

**Response**: We apologize for the misunderstanding. The current figure only considers sex; the previous label at the end of the document was incorrect. We have now updated the title to: Disability levels by sex according to the WHODAS 2.0 domains and EQ-5D-5L dimensions. Note: Bars and error bars represent, respectively, the means and standard deviations. We have also revised the figure to include the four bars that were previously missing.

**Multivariable analyses**

46. Line 415: ‘MD: mild disability’ is given as part of the caption of Figure 3, but when looking at Figure 3, I can only find ‘AMD’. Is this ‘Absent/missing data’? If so, the caption needs to be corrected. Also, when listing the Figure captions in Lines 796 to 802, the captions for Figures 3 and 4 are not given.

**Response**: We corrected the caption with AMD: Absent/missing data; MD: mild disability. We corrected all the Table and Figures information at the end of the manuscript when necessary.

47. Line 417: Delete ‘Last’, so it reads ‘Only sex, age, …’

**Response**: Done.

48. Line 418: Add ‘values’ after ‘adjusted Odds-Ratio (aOR)’ so it reads ‘adjusted Odds-Ratio (aOR) values were …’.

**Response**: Done.

49. Line 421: Add ‘the’ before ‘model’ so it reads ‘Regarding the model ...’.

**Response**: Done.

50. Line 423: Change ‘significantly retained in the final model’ to ‘statistically significant and, therefore, retained in the final model’.

**Response**: Done.

**EQ-5D-5L results Descriptive results**

51. Line 441: Delete ‘and’ after the semicolon.

**Response**: Done.

52. Lines 439 and 442: Has rho been defined? See comment above.

**Response**: Done, see above response.

**Discussion**

53. Line 513: Change ‘One should perhaps interpret this item with caution while waiting further …’ to ‘This item should be interpreted with caution while awaiting further ….’.

**Response**: Done.

54. Line 515: Re-write ‘It is regrettable that Kato-Katz was used instead of more sophisticated techniques for diagnosing intestinal nematodes or protozoa, making it possible that a number of infections could not be identified.’ I suggest: ‘Kato-Katz was the only method used for diagnosing intestinal infections, hampering our ability to detect other potential (protozoan and/or nematode) infections.’

**Response**: We thank the reviewer for this suggestion.

55. Line 517: Add ‘status’ after ‘*T. trichiura* infection’, so it reads ‘*T. trichiura* infection status’, as for this parasite infection intensity was not measured.

**Response**: Done.

56. Lines 524-525: Re-write ‘For example, it has been shown in a murine model infected with *T. muris* an increase in anxiety’ to read ‘For example, an increase in anxiety has been shown in a murine model of *T. muris* infection’.

**Response**: We thank the reviewer for this suggestion.

57. Line 529: Change to ‘did not show statistically significant associations’.

**Response**: Done.

58. Line 532: Replace the full-stop after Table 2 with a comma, so it reads ‘(Table 2, aIRR = 1.23)’. Lines 542-543: Delete the determinate articles so it reads ‘were significantly associated with *L. loa* MFD but not with history of Ew episodes’.

**Response**: Done.

59. Line 553: Add ‘detrimentally’ so it reads ‘was found to detrimentally impact mobility’. Line 554: Change ‘poxy’ to ‘proxy’.

**Response**: Done.

60. Line 566: Delete ‘*Loa*’ or change to ‘*L. loa* MFD’. Line 567: Replace ‘impacted’ by ‘affected’.

**Response**: Done.

61. Line 569: Should ‘associated with microfilariae’ be ‘associated with *L. loa* MFD’? Line 575: Change ‘Conversely’ to ‘By contrast’.

**Response**: Done.

62. Line 579: Delete ‘the’ so it reads ‘associated with loiasis status’. Line 579: Replace ‘conversely to’ with ‘in contrast to’.

**Response**: Done.

63. Line 593: Do you mean considerable disability rather than significant disability?

**Response**: We replaced with meaningful.

64. Line 616: Delete ‘Finally’ so it reads ‘While other potential explanatory factors’. There is a ‘Lastly’ in Line 620, so this cannot be ‘Finally’.

**Response**: Done.

**Acknowledgements**

65. Line 652: Spell out PNLO or add ‘(PNLO)’ to line 19 (in affiliations).

**Response**: Done.

**Figures**

66. Lines 796-802: Titles and captions for Figures 3 and 4 are missing.

**Response**: Done.

**Supplementary Materials**

67. The list seems incomplete or incorrect, as in the Main Text ‘S6 Figures’ and ‘S10 Figures’ are mentioned, but not listed? A suggestion to the authors (and in line with PLoS Negl Trop Dis guidelines) is to prepare a single Supplementary S1 File, and within it, name the items using letters. For example, Text A, Text B, Table A, Table B, Figure A, Figure B, etc. This will also avoid confusion with the items of the WHODAS questionnaire, that also start with an ‘S’. To illustrate this, in Line 192, where it says ‘see appendix S1 Questionnaire’, you would say ‘see S1 File, Questionnaire A’ for the WHODAS questionnaire, and ‘S1 File, Questionnaire B’ for the EQ-5D-5L questionnaire (Lines 213-214). This will also help the reader to have all supplementary information items in one single Supporting Information S1 File.

**Response**: We thank the reviewer for these helpful comments. Current guidelines recommend using the letter “S” followed by a number (e.g., S1, S2) to refer to each item of supplementary material. In this revised version, to improve clarity and reduce potential confusion with the labeling of questionnaire items, we now refer to supplementary materials as “S1 Appendix,” “S2 Appendix,” etc., in the main text. Additionally, we now spell out questionnaire items (e.g., “question 5”) rather than using the “S” prefix (e.g., “S5”), resulting in clearer references such as “question 5 of the S2 Appendix. And we corrected all missing information regarding figures and their legends.

**REVIEWER 4:**

1. The objectives and methods are clearly stated. The population is well described and appropriate to the hypothesis. The samples size is adequate. No concerns about ethical requirements

**Response**: We thank the reviewer for this comment.

2. These appear to be post-hoc analyses. The tables are overly detailed and difficult to follow. Some have text far too small to be legible. There is an over-reliance on acronyms in the text of the manuscript that makes it very difficult to follow. For example, FD, PD, MB, MBP, HbAS, aIRR, etc. It would be clearer if the actual terms were used, especially where there may be confusion.
   The figure legends are insufficient. In particular, figure 2 is unclear--do black and grey represent male and female or NO history of eye worm vs >10 episodes of eye worm? If the latter, the intermediate group (1-9 episodes of eye worm) should also be shown. Captions for figures 3 and 4 are missing (as are captions for the figures in the appendix).

**Response**:

- We did not conduct any post-hoc analyses. The analysis of these questionnaires was performed according to our pre-specified analysis plan and following the recommended guidelines: Ustun TB, Kostanjsek N, Chatterji S, Rehm J. Measuring Health and Disability - Manual for WHO Disability Assessment Schedule WHODAS 2.0 (World Health Organization, 2010), and The EuroQol Group (1990). EuroQol - a new facility for the measurement of health-related quality of life. Health Policy, 16(3):199-208.
- We agree with the reviewer and have increased the font size in Tables 1 and 2 (with slight formatting adjustments to Table 2 accordingly).
- We also removed several abbreviations (e.g., MD, PD, FD, SCD) to reduce the overall number of abbreviations, improve readability, and minimize potential confusion.
- We apologize for the issue with Figure 2. Although the updated figure was included, its title and legend had not been revised. We have now corrected this and ensured that all figures are labeled and captioned.

3. Many of the conclusions are based on conjecture rather than data. For example, the primary message of the paper is that poorer disability or quality of life scores are associated with having >10 eye worm episodes. Poorer scores are also highly correlated with increasing age. Yet there is no discussion of the relationship between having >10 eye worm episodes and increasing age. Obviously, the longer one lives and is infected, the more eye worm episodes one will experience. Does the correlation with eye worm episodes simply reflect the age of those with >10 episodes?

**Response**: We thank the reviewer for raising this potentially important point. However, we carefully assessed this issue and found no statistically significant association between the total number of eye worm episodes and age (Chi² p = 0.283), nor any meaningful correlation to support such a relationship (Cramer’s V = 0.0848, which is extremely low). Furthermore, we applied a multivariable model, which by design accounts for potential confounding factors, including age. Based on our results, we can state—not merely hypothesize—that, at equal age, the number of adult worms experienced over a lifetime has a statistically significant association and effect on some of our outcomes. As already mentioned in the Methods section, “The correlations between each explanatory variable were evaluated using the Spearman’s coefficient (for continuous variables) or the Cramer’s V test (for categorical variables).” We have now added the following sentence to the Results section: “No statistically significant association was found between age and the number of eye worm episodes (Chi² p=0.283; Cramer’s V value of 0.0848), suggesting that the number of episodes does not increase with age.” Finally, in the Discussion section, we added the sentence: “The number of past Ew episodes was found to be associated with worse mobility scores, independently of age.” Here is the percentage by Ew categories according to our age variable:

| Age | 0 Ew | 1-5 Ew | 6-10 Ew | >10 Ew |
| --- | --- | --- | --- | --- |
| 18-28 y.o. | 32 (59.3%) | 13 (24.1%) | 7 (13.0%) | 2 (3.7%) |
| 29-38 y.o. | 68 (62.4%) | 21 (19.3%) | 12 (11.0%) | 8 (7.3%) |
| 39-48 y.o. | 87 (55.8%) | 31 (19.9%) | 21 (13.5%) | 17 (10.9%) |
| 49-58 y.o. | 102 (46.1%) | 47 (21.3%) | 49 (22.2%) | 23 (10.4%) |
| 59-68 y.o. | 91 (49.5%) | 42 (22.8%) | 32 (17.4%) | 19 (10.3%) |
| >68 y.o. | 45 (48.4%) | 18 (19.4%) | 20 (21.5%) | 10 (10.8%) |
| Total | 425 | 172 | 141 | 79 |

4. In the abstract and introduction, the word "would", which connotes a hypothetical or counterfactual state, is used in situations not intended to be counterfactual (i.e. "would be infected", "would be a frequent reason", "would mainly affect daily activities", etc. Consider revision.

**Response**: We thank the reviewer for this comment, we modified the words “would’.

5. Figure 1 "126 excluded (<500 microfilaremia per mL) is nonsensical. Microfilaremia is a condition, not a parasite stage.

**Response**: Done. We thank the reviewer for their careful and thoughtful review.

6. line 349 "none significant interaction"

**Response**: We replaced none with no statistically significant interaction.

7. This manuscript is a useful attempt to further define the effect of loiasis on disability and quality of life among those infected. It employs reasonable methods and the statistical analyses seem sound. However, there are some aspects that make it difficult to follow, and the conclusions are based on conjecture rather than data.

**Response**: dfdf

**Specific comments:**

8. These data do not show that adult worm burden causes the quality of life issues. They show a correlation with >10 eye worm episodes, which could be age-related as discussed above.

**Response**: See comment above — there is no association between the total number of eye worms over a lifetime and age. Moreover, we used a multivariable model that would have accounted for this potential confounder if it had existed. We have now added a few words in the text to clarify this point for readers who might have had a similar concern.

9. Some methods need further explanation: line 149: how were discrepancies in Mf count defined? Were the slides read twice by one technician or once each by two technicians?

**Response**: Inter-reader variability is indeed an important factor to consider. We have now added ‘(±20%)’ and specified that each TBS was independently read once by each technician.

10. Explanatory variables (line 240): Why was age a categorical variable instead of a continuous one? If there were 10 age categories used, why are only 6 shown in the tables and figures? Why were eye worm episodes categorical rather than continuous?

**Response**: We apologize for the unclear wording. As shown in the tables, age was categorized in 10-year intervals, and we have corrected the description in the Methods section. Regarding age, we chose to use a categorical approach. While it may seem intuitive to assume a linear increase in mobility-related impact with age, this may not hold true for other dependent variables, such as anxiety, daily activities, or social relationships. To ensure consistency and avoid using different approaches for analyzing age across outcomes, we opted for a categorical rather than a continuous variable. Regarding eye worm history, due to the high number of missing data (173 individuals, i.e., 17.5%), and in order to retain information on the other covariates, the only viable option was to categorize this variable. Using it as a continuous variable would have led to the exclusion of 17.5% of the total sample from the models.

11. There seem to be 51 or more degrees of freedom in the multivariate model (including 10 age categories). Is this appropriate? Why not conserve degrees of freedom by analyzing the appropriate input variables as continuous rather than categorical?

**Response**: See our previous response.

12. Why convert the WHO-DAS-2 scores to a scale of 100 rather than using the actual, validated score? Was this done simply by multiplying by a conversion factor? If so, why was this necessary? If there is a good rationale and the scale of 100 scores are retained, they should at least be referred to as "adjusted" or "modified" WHO-DAS-2 scores.

**Response**: We followed the scaling recommendations on a 0–100 scale, as indicated in the references cited at the beginning of each questionnaire’s presentation. This normalization facilitates the use of standard cut-offs, such as 25 and 50, which correspond to moderate and severe levels of disability, respectively. Additionally, expressing disability levels as percentages (i.e., out of 100) improves readability and interpretability for readers. We have added the reference to Üstün et al. after these statements in the Methods section.

13. line 407 "we estimated" suggests that the numbers presented are a guess. Why not simply report that the model-adjusted scores WERE as reported?

**Response**: Done.

14. Do Table 2 and Table 3 show ALL the variables included in the multivariable analysis?

**Response**: We thank the reviewer for this clarification. We have added “Final” at the beginning of the title. These are indeed the final models, as described in the Methods section. The full (saturated) models including all variables are presented in the supplementary material (as indicated in the main text with for example (S8 and S9 Appendix for saturated models).

15. Could the association with reported episodes of eye worm simply represent a tendency on the part of some individuals to over-report symptoms?

**Response**: We thank the reviewer for raising this point. It is indeed possible that participants may associate the eye worm with joint pain or daily discomfort—potentially based on misconceptions or false beliefs. We cannot entirely rule out this effect; if present, it would imply the existence of a differential bias that could affect our results. However, we conducted an ethnological study in our study area. While 30% of participants did associate Calabar swelling with joint pain, residents most commonly linked eye worm episodes with conjunctivitis and reduced visual acuity, without mentioning any connection to joint pain or mobility issues. These findings are from ongoing research currently being prepared for publication, and thus cannot be cited directly. Nevertheless, we have added the following sentence to the limitations section: “Third, we cannot entirely rule out the possibility of a subtle differential bias whereby individuals experiencing more Ew episodes may associate this symptom with joint pain or daily discomfort, potentially influencing our results. However, we believe such bias to be minimal, as informal discussions with local residents suggest that Calabar swellings should be more commonly associated with several of our indicators, whereas Ew episodes should be primarily linked to reduced visual acuity.”

16. Line 483. What basis is there for the claim that WHO-DAS-2 is particularly suited for rheumatologic conditions? If there is support for this claim, it should be cited.

**Response**: We thank the reviewer for this thoughtful comment. Although the WHODAS 2.0 is a generic disability assessment tool applicable across a wide range of conditions, it has been widely used and validated in rheumatologic diseases such as rheumatoid arthritis and osteoarthritis, making it particularly informative in these contexts. However, even though it has indeed been more frequently used in these conditions (and may therefore offer better established validity in such cases), it is inaccurate to state that it is “particularly adapted” to them, as this would imply greater suitability compared to other conditions. We have therefore removed this sentence.

17. Line 500: "It appears that factors related to....were only marginally affected." Affected by what? Is there a comparison here, or are you just saying that scores in these areas across the whole study population were normal?

**Response**: There is no direct comparison, but as shown in the tables and Figure 2, the levels of disability for these specific scores are clearly lower than for the others. We have therefore rephrased the end of the sentence as follows: “Scores related to the ability to take care of oneself (“WHODAS self-care” and “EQ-autonomy”) and to social participation (“WHODAS social”) were generally low across the study population, suggesting that these aspects of functioning were only minimally impacted.”

18. The discussion theorizes that adult worms are causing mobility impairment, but if >10 eye worm episodes are found only in older individuals, this may have nothing to do with worms and may only represent age. An analysis of the correlation between eye worm episodes and age would be helpful. Line 553: "the number of past Ew episodes was found to impact mobility" is incorrect, since it assumes causation, which is not proven. Ew episodes were only correlated with mobility. Same issue line 580. Finally, the conclusion that "adult worms primarily affect daily activities through peripheral symptoms, such as joint-related discomfort (with notable mobility impairment)" is not warranted. Again, this study shows association, not causation, and this statement should be revised.

**Response**: As we already mentioned earlier, there is no association between the number of eye worms and age. We have added a brief sentence in both the Results and Discussion sections. The new sentence in the abstract is: “Our findings strongly suggest that adult worms may primarily impact daily activities through peripheral symptoms, such as joint-related discomfort with notable mobility impairment, which could contribute to mobility impairment”.

**REVIEWER 5 (called reviewer 5 but attached file called reviewer 3, we think this is the same reviewer: see all the detailed responses of the attached file in the reviewer 3 section).**

Thee Methods have been reviewed by previous referees and I have no further comments on these.

The Figures need some attention and I have described the issues in the attached file.

I have made a number of editorial suggestions listed in the attached document.

This is an important paper that had already been reviewed by two competent referees. I read the revised manuscript and the replies to referees by authors. I found that the authors addressed carefully and point-by-point the referees' comments and queries. The Figures and Supporting Information need attention. I also suggest that 3-4 references be added. I have made editorial suggestions in an effort to improve the clarity and conciseness in places, as well as as the grammar where necessary.

**Response**: As mentioned, we believe there may have been a labeling issue, and that Reviewer 3 and Reviewer 5 are actually the same person. We would like to once again thank this reviewer for their careful and detailed review, which has helped improve our manuscript. All our responses can be found under the section for REVIEWER 3.
