# Supplementary material for: Disability and quality of life assessment using WHODAS-12 items 2.0 and EQ-5D-5L in a rural area endemic for loiasis in the Republic of Congo: A population-based cross-sectional study (the MorLo project)
Source: PLoS Negl Trop Dis. 2025 Sep 15;19(9):e0013491. doi: 10.1371/journal.pntd.0013491 (PMC12449028; doi:10.1371/journal.pntd.0013491)
Supplement: Attachment — Submitted filename: Response_to_reviewers_WHODAS_R3.docx [file pntd.0013491.s017.docx]

**Responses to reviewers PNTD-D-24-01363R2**

**Disability and quality of life assessment using WHODAS-12 items 2.0 and EQ-5D-5L in a rural area endemic for loiasis in the Republic of Congo: a population-based cross-sectional study (the MorLo project)**

**Reviewer #6:**

**Tables and figures comments:**

1. Table 1:

-would be clearer if you had vertical line separating data columns for WHODAS vs EQvts vs EQvas
-there should be further discussion of statistical differences between the data shown ex. Differences in the different age groups for each type of score

**Response**: We thank the reviewer; we added vertical lines in this Table (now Table 2). And we have added additional sentences in the discussion, especially for sex. Regarding the role of age, we have slightly expanded our discussion to better reflect the consistent gradient observed across both WHODAS and EQ-5D-5L scores. However, as previously mentioned in response to another comment, Table 1 presents descriptive data without statistical testing. Our interpretation is therefore primarily based on the results of the multivariate models, which offer robust evidence to support a concise and targeted discussion on age-related differences.

2. Most figures need further explanation in the legends.

**Response**: We sincerely thank the reviewer; we added more explanation.

3. Fig 2: explain light vs dark gray color for the two bars (assume one is male and one female)
Explain what is on the Y axis (% disability) and how it was calculated. I could not find this in the methods. I at first assumed this was the % of the summed global score from each domain, but the global score was also only 20-30%.

**Response**: We thank the reviewer for this point, which we had indeed forgotten to clarify. We have now added the explanation in the figure legend. These percentages represent the level of disability, first for the global score and then for each individual item or domain (according to WHODAS and EQ). The global score reflects the average disability across all WHODAS items; the other values (to the right) correspond specifically to the mean level of disability (expressed as a percentage) for each item or domain individually.

4. Table 2: why no aIRR for Sex and cognitive score?

**Response**: As explained in our method section, we performed models with a manual backward selection procedure using the global Wald test to retain only variables with p<0.100 in the final models and/or including variables with potential modality of interest (p<0.100). The variable sex was therefore not included in our final models, as its p-value was greater than 0.100.

5. Fig S7 is supposed to have the raw scores for each EQ dimension on scotherale of 1-5. However, listed are scores that range from 4-49? What is the mean listed in S7? This is unclear to me

**Response**: We thank the reviewer for noticing this error. As with the WHODAS, we used scores out of 100 in the descriptive section to obtain an easily interpretable percentage. These are therefore mean scores out of 100 for each of the six dimensions. We clarified this information in the Methods section and added a new Table (Table 1) to summarize all our scales for each score to facilitate the reading.

**Clarity and grammatical recommendations**

-Line 48: more typical to say associated with, rather than associated to

**Response**: We thank the reviewer; we did the change.

-Line 52-53: awkward sentence (notable mobility impairment contributing to mobility impairment)

**Response**: We thank the reviewer; we did the change.

-line 99-100: might be clearer as loiasis (defined as either microfilaremia or a history of adult worms, based on eye worm or Calabar swelling)

**Response**: We thank the reviewer; we did the change.

-Line 189: Awkward: recommend substituting “data points” for “informations”

**Response**: We thank the reviewer; we did the change.

-lines 214-218: would clarify here that you are measuring days with each level of disability (not a certain score indicating a level of disability as done elsewhere. Might be more clear if written: “They were asked to provide the number of days the difficulties were present (termed H1, indicating mild disability)…

**Response**: We thank the reviewer; we did the change.

-line 387-392: awkwardly written. Could change to The scores recorded for WHODAS 2.0 selfcare, WHODAS 2.0 social, and EQautonomy showed that these domains and dimension represented a very low proportion of disability. Whereas other parameters had a higher percent of disability (rather than a preoccupation)

**Response**: We thank the reviewer; we did the change.

-line 402: would clarify: number of partial disability days increased with the frequency of Calabar swellings

**Response**: We thank the reviewer; we did the change.

-Lines 471 and 473: would change to worse anxiety (or mobility) scores rather than impact on anxiety.
**Response**: We thank the reviewer; we did the change.

**General Comments**

1. It might be useful to specify the language or languages that informed consent was obtained in (line 125)
**Response**: We thank the reviewer. Written information was provided in Kikongo and Lingala, in accordance with the ethics committee’s recommendations, and explanations were given in the local vernacular language when needed for some participants. We have now added this information.

2. Line 190: Some background on Raploa might be useful for those outside the Loa field

**Response**: We have added the clarification in the text.

3. Line 366-373: should cite which figure presents this continuous variable data, or if the raw data is not presented in the paper, possibly another supplemental figure with a scatter plot showing score vs year of schooling could be added (with each different category in a different color)

**Response**: We added three figures in the supplementary material.

4. The text headings separate out WHODAS 2 and then later EQ-5D-5L for descriptive and multivariate analyses, but the text combines description of both WHODAS 2 and EQ-5D-5L.

**Response**: We sincerely thank the reviewer. We slightly modified when necessary.

5. Additional description of the raw data in table 1 would be useful. There is basically no description of the table 1 results, only the continuous results which are not presented in a figure or table.

**Response**: We thank the reviewer for this useful comment. As you noted in a later comment, this manuscript already contains a large volume of information in the text due to the number of outcomes and the nature of the analyses. We believe that Table 1 is sufficient to communicate the overall level of disability, and to describe the different domains or dimensions across the population. Our primary objective for Table 1 was to provide readers—especially researchers and healthcare professionals—with detailed values they can use as a reference, rather than to elaborate on these results again in the text. We are concerned that expanding the description of Table 1 in the main text would unnecessarily lengthen the manuscript without adding further value. However, to address your suggestion, we have included additional descriptive figures (new S5a, b and c) in the supplementary material. We are happy to follow the editor’s decision if these figures should be moved into the main manuscript.

6. Table 1 could benefit from statistics – there are only statistics for the multivariate analysis not the raw data in table 1. For example, it is interesting and useful to show no statistical difference in scores based on eosinophilia.

**Response**: We appreciate this observation. However, as noted in our previous response, the aim of Table 1 was to provide a detailed overview of the disability and quality-of-life scores to inform readers and practitioners. Including statistical comparisons for each subgroup (e.g., by eosinophilia status) would require numerous tests, which may not be appropriate given ongoing concerns in the statistical community regarding multiple comparisons and overinterpretation of unadjusted associations. We believe that the multivariate analyses—which account for potential confounding—are better suited to assessing these differences with statistical rigor. For this reason, we opted to keep Table 1 descriptive and reserve statistical testing for the adjusted models presented in the main results.

7. Paper could benefit from discussion of how Mf might change over time and skew results. Mf count was not repeated during 2022 enrollment and was based on 2019 assessment.
On further reflection, it is unclear if the TBS was done off blood from 2022 or the original 2019 assessment and which is listed in table 1.

**Response**: We apologize for the lack of clarity. The 2019 results were only used as part of the pre-inclusion screening; a new thick blood smear was performed for all participants in 2022. This has now been clarified in the text (see “Study design” sub-section).

8. Abstract conclusions focus most on mobility as does the concluding paragraphs, but Fig 2 demonstrates high scores for mobility, but also pain and anxiety and daily living. This should be further explained. I think it is also a little unclear what they mean by peripheral symptoms in the abstract

**Response**: We sincerely thank the reviewer. We revised the “conclusions” part of the abstract.

9. The main conclusion in the abstract talks about impact of adult worms (rather than EW episodes) on mobility. This has been raised by a previous reviewer. I think it should be stated more clearly that the main correlation between worse QOL scores is with number of eye worm episodes, which they are assuming to be a proxy for number of adult eye worms. It also only highlights mobility, even though their data seems to indicate issues with mobility, pain, anxiety and other concerns as well.

**Response**: We sincerely thank the reviewer. We revised the “conclusions” part of the abstract.

10. The paper overall is dense and difficult to read given the large number of different scores they are referencing and their very different scales. It is very helpful for there to be reminders of what each scale represents with each table, such that someone could read just the table and legend and understand the data presented. This would be redundant but I think overall helpful to the flow of the manuscript. The full description follows table 2 and 3 but not table 1 (should add WHODAS description to table 1)

**Response**: We thank the reviewer once again for all the suggestions that contribute to improving the clarity of the manuscript. This is also why we added Table 1, which summarizes the construction and use of our scores. We believe this table is helpful and may be sufficient without further detailing the additional footnotes, which we have nonetheless slightly expanded, as well as the figure captions.

11. I appreciate the good discussion of the surprising result that Mf count does not appear correlated with worse QOL, despite being correlated with organ disfunction and death. There is much written about the chronic eosinophilia in loiasis. I think there should be more discussion of the lack of correlation between eosinophilia and QOL, as well as how the cutoff of 2 was chosen.

**Response**: A cutoff of 2 g/L was chosen to increase the specificity of any potential associations with organ-related dysfunction, and thus with the broader impact on individuals as captured by quality of life (QoL) indicators. While lower thresholds are often proposed—particularly in cases of primary hypereosinophilic syndrome, where a large study identified 1.5 × 10^9^ cells/L as a more sensitive threshold for predicting complications (Bjerrum et al. "Association of the blood eosinophil count with end-organ symptoms." Annals of Medicine and Surgery 45 (2019): 11-18) —, parasitic-associated eosinophilia is generally considered less likely to result in severe complications. Therefore, to enhance specificity and because our sample size allowed it, we arbitrarily selected the 2 × 10^9^ cells/L cutoff. A brief note has been added in the Methods section (“Explanatory variables” sub-section) to clarify this choice. Regarding the lack of association between eosinophilia and QoL for most of our outcomes, we thank the reviewer for highlighting this point. We agree that this is a particularly interesting finding. For this reason, we reanalyzed our models to explore potential interactions between eosinophilia and the main parasitic infections. Corresponding additions were made at the end of the Methods section and in the Results section (see “Correlations, multicollinearity, and interaction terms” and “Multivariable analyses” sub-sections). In addition, a specific paragraph has been added in the Discussion.

The paper addresses an important clinical question: the impact of loiasis on overall wellbeing. This infection impacts millions of people and has been substantially neglected in terms of research into clinical course and treatment discovery. Further, several recent studies have demonstrated clear associations between loiasis and end organ damage and increased mortality. Therefore, this study looking at how loiasis impacts overall quality of life is clinically interesting and important. They also delve into which aspects of this parasitic infection are most correlated with wellness (ex: microfilaria or signs more associated with adult worms). However, the paper is overall difficult to read given the many different scoring systems used. Have made some suggestions to help improve clarity and presentation.

**Response**: We sincerely thank the reviewer. We hope that our revisions improve clarity and readability. We have also added a new table summarizing how the different scores were used, to facilitate understanding and follow-up throughout the manuscript.
